# Integration of the Transcriptome and Glycome for Identification of Glycan Cell Signatures

**DOI:** 10.1371/journal.pcbi.1002813

**Published:** 2013-01-10

**Authors:** Sandra V. Bennun, Kevin J. Yarema, Michael J. Betenbaugh, Frederick J. Krambeck

**Affiliations:** 1 Department of Chemical and Biomolecular Engineering, Johns Hopkins University, Baltimore, Maryland, United States of America; 2 ReacTech Inc., Alexandria, Virginia, United States of America; 3 Department of Biomedical Engineering, Johns Hopkins University, Baltimore, Maryland, United States of America; National University of Singapore, Singapore

## Abstract

Abnormalities in glycan biosynthesis have been conclusively linked to many diseases but the complexity of glycosylation has hindered the analysis of glycan data in order to identify glycoforms contributing to disease. To overcome this limitation, we developed a quantitative N-glycosylation model that interprets and integrates mass spectral and transcriptomic data by incorporating key glycosylation enzyme activities. Using the cancer progression model of androgen-dependent to androgen-independent Lymph Node Carcinoma of the Prostate (LNCaP) cells, the N-glycosylation model identified and quantified glycan structural details not typically derived from single-stage mass spectral or gene expression data. Differences between the cell types uncovered include increases in H(II) and Le^y^ epitopes, corresponding to greater activity of α2-Fuc-transferase (FUT1) in the androgen-independent cells. The model further elucidated limitations in the two analytical platforms including a defect in the microarray for detecting the GnTV (MGAT5) enzyme. Our results demonstrate the potential of systems glycobiology tools for elucidating key glycan biomarkers and potential therapeutic targets. The integration of multiple data sets represents an important application of systems biology for understanding complex cellular processes.

## Author Summary

Glycans are the sugar attachments that are found on proteins and lipids. These highly variable and structurally diverse sugar chains confer distinctive characteristics to the cell surface. Recent research has revealed that these glycan profiles can represent important signatures of disease states and thus understanding glycan processing and structures in cells is an important systems biology goal. Glycan structures are often characterized through mass spectral analysis while their glycosylation processing enzymes are characterized using gene expression profiling. Unfortunately, due to the complexity of glycosylational processing, it has been difficult to relate these disparate data sets until now. In this paper we demonstrate for the first time the ability of a systems glycobiology model to link glycan structural data obtained from mass spectral analysis with mRNA expression data in terms of enzyme activities catalyzing the glycosylation reactions in the cells. We show that such a systems biology model enables identification of distinctive and subtle glycan fingerprints differences between prostate cancer cell stages (androgen-dependent and more metastatic androgen independent). This systems approach will enable us to use high throughput glycomics and gene expression data sets in order to specify glycan-based signatures as important diagnostic markers of disease and potential therapeutic targets.

## Introduction

Glycosylation, a broad term covering the addition of oligosaccharides (glycans) to proteins and lipids followed by their subsequent modification during transit through the secretory apparatus, is an intricate intracellular process whose complexity hinders ready interpretation from mass spectral and other data sets. Nonetheless, three decades of research has made it clear that the glycosylation of healthy and diseased cells often diverges resulting in glycan changes that contribute to pathological progression [1], [2], [3], [4], [5]. A prime example of the contribution of glycan analysis to the understanding of a pathological process and the development of clinically relevant biomarkers is provided by prostate specific antigen (PSA) [6], [7], [8], [9], [10]. Changes in the glycosylation status of this widely used biomarker for prostate cancer screening have been useful in improving its specificity and ability to distinguish benign forms of this disease from highly malignant cancer [11], [12].

While considerable progress has been made from decades of painstaking research focused on PSA, efforts to identify additional glycan markers of disease suffer from the difficulties in identifying specific glycosylation changes. However, with the current proliferation of high throughput ‘*omics*’ approaches, opportunities are at hand to develop and implement methodologies that analyze the resulting large data sets in order to provide critical glycan signatures of disease; for example to expand analyses from PSA to additional prostate cancer biomarkers and, more broadly, from prostate cancer to the numerous cancers and diseases known to have abnormalities in glycosylation. Unfortunately, the disparate sets of data needed to fully characterize glycosylation –including expression profiles of the enzymes involved in glycosylation, the activities of the resulting enzymes, and finally the large number of glycans actually produced by these enzymes – cannot be directly compared and there is yet no facile way to integrate the data to generate meaningful biological insights.

Transcriptional profiling of mRNA allows quantitative global assessment of the many genes involved in glycan biosynthesis i.e. glycosyltransferases, the enzymes responsible for generating glycans. A wealth of data is also becoming available from the detailed assessment of the glycans using mass spectrometric techniques [13]. Despite progress on both ‘*omics*’ fronts, useful bioinformatics tools to identify glycan structural data and also to link these findings with transcriptional profiles of the enzymes that produce these sugars have lagged. For example, a common approach for mass spectrometry-based glycoprofiling involves a one-to-one data base matching of particular mass spectrometry measurements to specific glycans from a known glycan library in order to annotate the mass spectra [14], [15]. Statistical database-driven approaches have attempted to relate gene expression levels to the abundance of specific glycan linkages [16], [17], [18]; however these approaches do not provide quantitative predictions of detailed glycan distributions. As a consequence, there is no clear understanding of how mRNA levels relate to the actual amount and distribution of glycans found within a healthy or diseased cell. In addition, current bioinformatic techniques only consider each mass spectral peak in isolation and do not consider other relevant peaks when making an identification or quantification.

In this work, we address this void with a novel systems biology model that interconnects glycan structural data obtained from mass spectrometry with changes in gene expression obtained from mRNA profiling of the relevant glycan processing enzymes. The glycobioinformatics approach interprets mass spectral data in terms of the activity of glycan-processing enzymes and then compares these values to those indicated from gene expression profile. The model identifies a number of unrecognized glycan structures and their abundances from mass spectral peaks by analyzing the entire mass spectrum in concert instead of considering each mass peak in isolation. The model can also process the relative enzyme transcript gene expression levels, and translates them into a synthetic mass spectrum and a quantitative glycan profile. This model approach has been applied to uncover subtle differences in the glycan signatures between two sets of cancer cells, specifically low and high passage, androgen dependent and independent (respectively) LNCaP prostate cancer cells. This effort has yielded insights into glycan-specific changes associated with malignant progression in this disease. In addition, this model approach enables a comparison of the result from the two *‘omics’* platforms and enables identification of consistent and inconsistent patterns across the two media. Moreover, this systems biology methodology allows users to gain insights into the complex multi-step cellular glycosylation process from disparate data sets and will serve as a critical step along the path towards the identification of key glycan biomarkers and therapeutic disease targets.

## Results

### Glycosylation model integration of gene expression and mass spectrometric data

**Figure 1:**
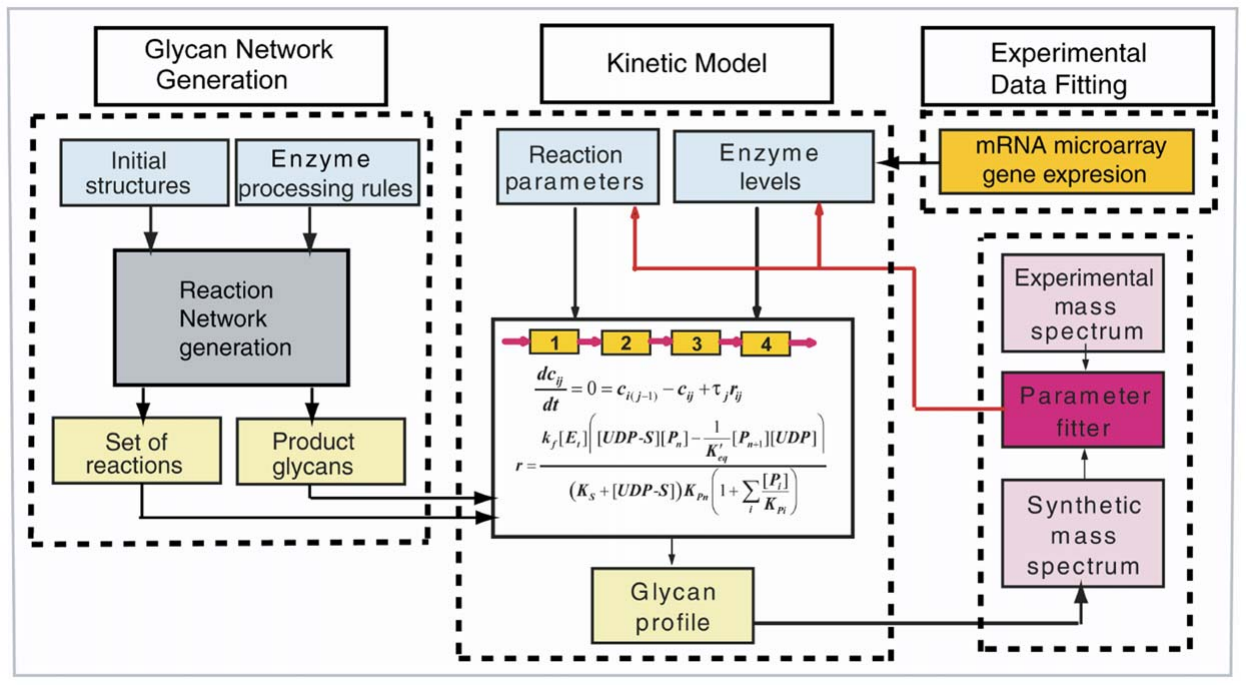
Schematic representation of the N-glycosylation model. The N-Glycosylation model generates a detailed annotated mass spectrum from the glycosylation reaction network by integrating three processing modules: 1-Glycan Network Generation, 2-Kinetic Model, and 3-Experimental Data Fitting. The Network Generation module uses reaction rules that express enzyme specificity and are applied in the beginning to the initial glycan structures (Man9 and Man8) in order to generate a set of reactions and subsequent product glycan structures. Next, the Kinetic Model module –where the Golgi apparatus is modeled as 4 well mixed reactors with a set of enzymes distributed through them- is solved for any set of enzyme concentrations and reaction rate parameters. These parameters include turnover numbers and dissociation constants for substrate and donor cosubstrate. Solving the model allows prediction of the complete profile and abundances of the glycan structures obtained from the generated glycosylation reaction network. In the last module: Experimental Data Fitting, a synthetic mass spectrum is obtained from the abundances of the significant glycan structures predicted by the model. This synthetic mass spectrum is compared with the experimental MALDI TOF mass spectrum by a non-linear fitting algorithm that solves the model multiple times by adjusting enzyme concentrations and other parameters each time. The ratio of the relative enzyme transcript gene expression levels can be processed by the model and translated in terms of a synthetic mass spectrum and a quantitative glycan profile. The model outputs are optimized until good agreement is achieved between the calculated glycan distributions expressed as a synthetic mass spectrum with the MALDI TOF experimental mass-spec.

In previous publications we applied a comprehensive mathematical model that incorporates a kinetic network for enzyme processing of N-glycans to interpret mass spectral and other glycan analytical data (HPLC) in terms of detailed glycan structures as well as specific enzyme activities [19], [20]. This analysis was useful for screening differences in glycan profiles and enzyme activities between different cell types. In this study we present an integrative glycan systems modeling approach that considers mRNA gene expression profiles for the glycosyltransferases and other enzymes involved in glycan synthesis together with matching MALDI TOF (Matrix assisted laser desorption ionization time of flight) mass spectral data. This data integrative modeling approach provides a thorough characterization of the changes in the glycan structural profile and abundances through the mass spectra. Model sizes used in this study are typically limited to about 10,000 to 25,000 glycan structures based on the implementation of a molecular mass cutoff and a network pruning method. This allows prediction of the complete glycan profile and its abundances for any set of assumed enzyme concentrations and reaction rate parameters. A schematic representation and explanation of how the model integration of mass spectrometric and gene expression data works is shown in Figure 1 (for more details see Materials and Methods).

### MALDI TOF glycoprofiling of high and low passage LNCaP cells

**Figure 2:**
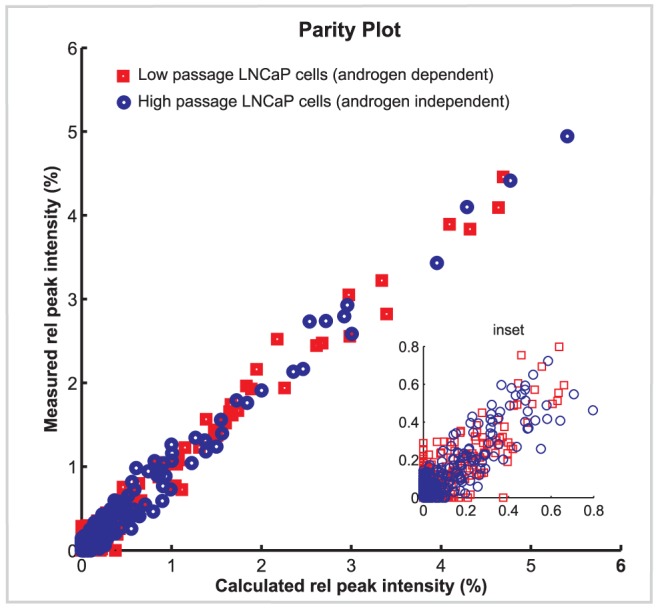
Parity plot. Shows fitting agreement between measured mass spectra of glycans from LNCaP high passage human prostate cancer cells (blue circle) and LNCaP low passage human prostate cancer cells (red square) with synthetic mass spectra calculated from the model. The plot includes mass numbers from 1400 to 4000 and has an associated RMS error of 0.05.

**Figure 3:**
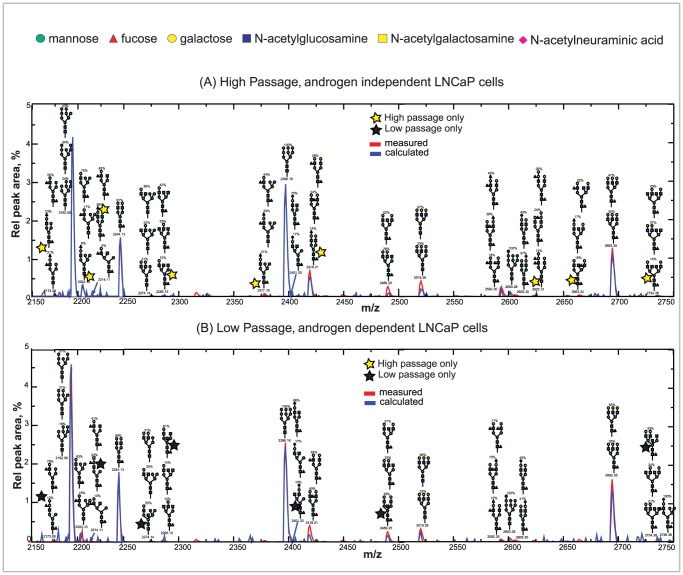
Comparison of model calculated synthetic mass spectra with measured spectra. Panel (A) High passage, androgen independent LNCaP cells at the top and panel (B) Low passage, androgen dependent LNCaP cells at the bottom. The plots show the mass range from 2150 to 2750 (full range modeled m/z 1400–4000). Units on the y-axis are relative intensities as % of total peak area of the spectrum in the modeled range. Units on the x-axis are the average m/z value of the peak. Each “peak” in the figure is the envelope of the isotopic satellite peaks of a single signal, resulting from the characteristic atomic content of a set of isomeric molecules.

High and low passage LNCaP cells provide a model for cancer progression from the androgen-dependent to the androgen-independent state [21]. The MALDI TOF mass spectrometry data for the low and high passage human prostate LNCaP cells are available at the Consortium of Functional Glycomics (CFG) database [22] and under supplementary material in Dataset S1 and Dataset S2. The C-33 cells, or low passage cells, include cells between passages 25 and 35 and serve as a model for androgen-dependent cells. The C-81 cells or high passage type were derived from the low passage cell line and have diverged into an androgen non-responsive state; they include cells between passages 81 and 125 [21]. The comparison of model generated synthetic mass spectra to experimental MALDI TOF mass spectra (Figure 2 and Figure 3) requires processing of the raw mass spectra, including baseline correction, mass calibration adjustment, peak integration and filtering of isolated spikes (individual peaks without isotopic satellites), software development for that end is described in Materials and Methods. Fitting of both MALDI TOF experimental data sets to synthetic mass spectra obtained through solving our N-glycosylation computational model as described in Figure 1 generates a set of glycan structures and abundances. The parity plot in Figure 2 gives the agreement of the calculated and measured experimental mass spectrometric data in the range of 1400–4000 for both high and low passage LNCaP cells. Peaks in agreement are located on the parity line; in general, a good fit is obtained for many of the glycans. The experimental mass spectrum extends to 5000; however, for this set of data few molecular masses are significant in the 4000 to 5000 range and thus the model was limited to the 4000 range.

This model approach can be readily implemented to assign a group of glycans with specific details on their associated structures and abundances to each peak in the MALDI TOF mass spectra of diverse mammalian cell types. For example, the good agreement obtained between the measured and synthetic mass spectra obtained from the model as indicated in Figure 2 for both LNCaP cell lines, is translated in Figure 3 as a close alignment of the experimental mass spectral levels (blue line) with the model predictions (red line) for most of the peaks. Overall, in Figure 3, we present a selected portion of the mass spectra for the low and high passage cell lines in the range of 2150 to 2750 with the dominant glycan structures producing each peak indicated by schematic structural diagrams. The comparative glycoprofiling of both cell lines is discussed in the next section and the complete set of glycans annotated across the 1500–4000 MS range is provided in Figure S1.

### MALDI TOF comparative glycoprofiling of high and low passage LNCaP cells

**Figure 4:**
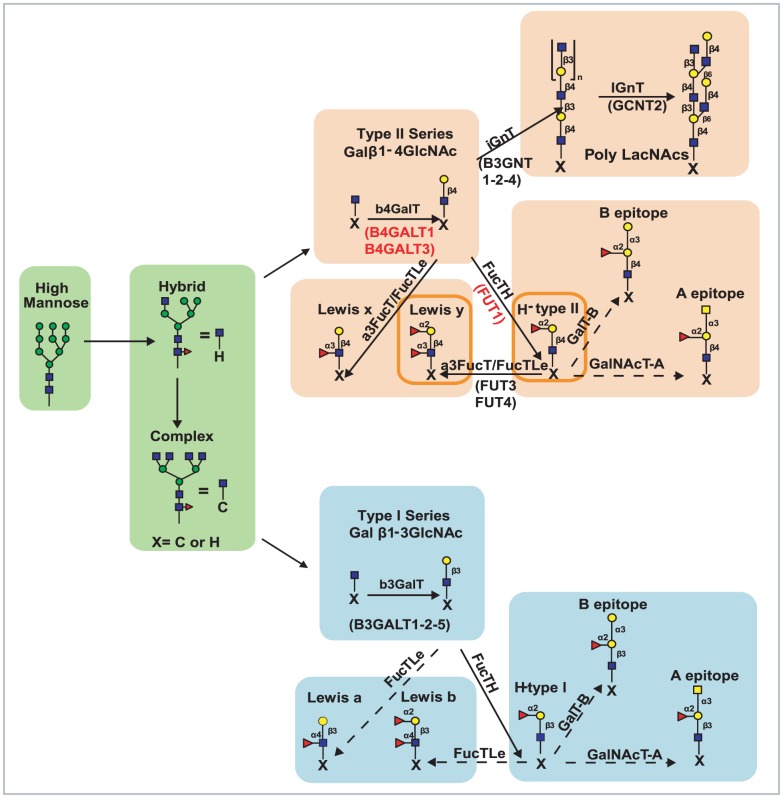
Schematic N-glycosylation pathway representation characteristic of high and low passage LNCaP cells. The steps to elaborate the glycan structures corresponding to both LNCaP cells lines are represented in a simplified N-glycosylation pathway according to the mass spectral structural data as well as the transcription expression data. A main feature for this pathway is the lower levels of Type I glycans (light blue filled rectangles) compared to type II glycans (light orange filled rectangles) in both cell lines, implying that glycans characteristic of both cell lines are principally type II glycans. Where indicated, genes in the pathways are listed in parenthesis and located below their corresponding enzymes. For example, the enzyme b4GalT, associated with type II glycans, is mainly represented by expression of B4GALT1 and B4GALT3 genes among other genes. The main difference between low and high passage LNCaP cell lines is the increased expression of FucTH (FUTI) in high passage LNCaP cells as noted in both microarray data and mass spectra based model predicted enzyme levels. This is also translated in increases of H(II) and Le^y^ epitopes (indicated by the glycan structures within the dark orange border rectangles). The dashed arrows point to glycan structures that are absent or marginally present. Quantitative detail of the corresponding type I and type II glycan abundances for the structures in this figure are depicted in both Figures 5 and 6. Initial steps of glycan formation as well as sialylation processing are omitted for simplicity.

High and low passage LNCaP cells that provide a model of cancer progression from the androgen-dependent to androgen-independent state exhibited considerable similarity in N-glycan patterns (Figure 3); this result was expected because both cell lines come from the same progenitor and only differ in the number of passages. However, the comparative glycoprofiling shown in Figure 3 establishes that there are also several differences between the two cell lines. For example, at the peak envelope starting at a monoisotopic mass of 2418.21 (the lowest mass of the isotope group), the high passage cells have about twice the signal of the low passage cells, partially due to the appearance of a structure containing the blood group H(II) epitope (Fucα1,2Galβ1,4GlcNAcβ) in the high passage glycans (See Figure 4 for N-glycan processing diagram). Another peak envelope starting at a mass of 2592.30 is about four times higher for the high-passage cells, due to the increased abundance of terminal fucose groups (Fucα1,2Galβ).

**Figure 5:**
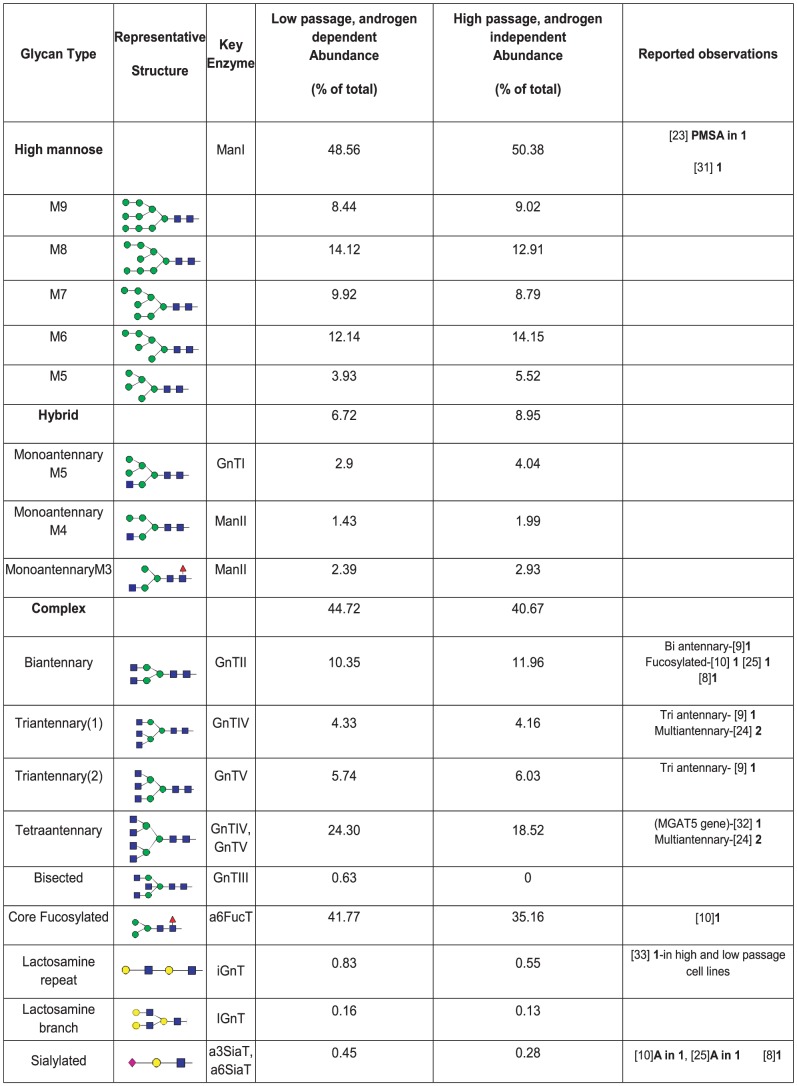
Abundance of N-glycans by type from model matching of MALDI-TOF data. Model predicted glycan abundances from MALDI TOF are listed together with reported literature data on the alteration of glycan processing in prostate cancer, especially trends in N-glycosylation for LNCaP cells. Reported literature are noted as: **1** LNCaP cells (mostly from PSA) unless indicated; **2** Prostate cancer tissue; **3** Seminal fluid; **4** Serum; **5** Other cell lines; **6** Metastases from human prostatic carcinoma; **A** Absent. High mannose glycans with 9 and 8 mannose residues (M9 and M8) leaving the endoplasmic reticulum (ER) are modified in the Golgi by the action of ManI to produce glycans with fewer mannose residues down to Man5. Hybrid glycans are formed by the action of enzymes GnTI, ManII and α6FucT on Man5 glycan structures. Complex glycans are formed by the addition of N-acetylglucosamine to form bi, tri and tetra antennary glycans that can undergo further modifications (Figure 6).

**Figure 6:**
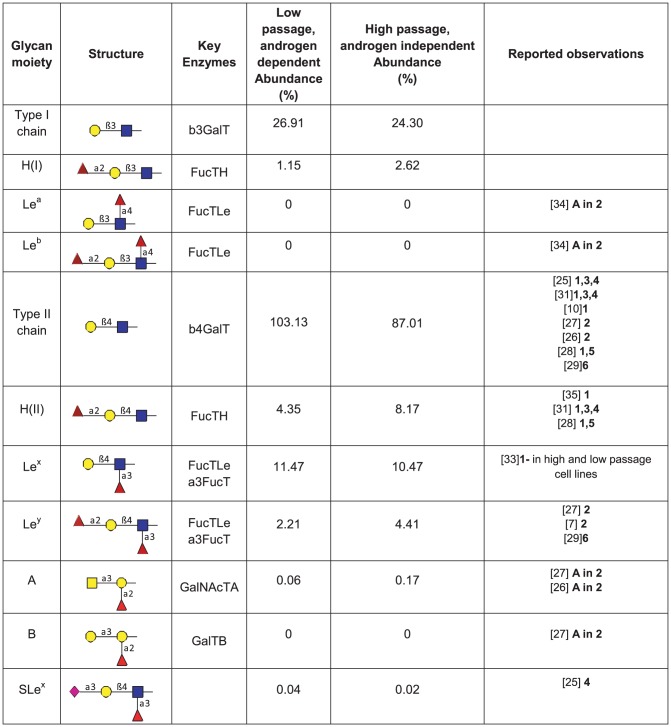
Type I and Type II Glycan moieties from model matching of MALDI-TOF. Blood groups can be classified based on the glycan moieties on the cell surfaces, the percentage abundance of different glycan moieties on low passage, androgen dependent and high passage, androgen independent LNCaP cells are included. Glycans with the moiety (Galβ1,3GlcNAc) are of type I. Further sugar additions to the branches of type I structures result in mature glycans with characteristic epitopes such as Lewis a, Lewis b, H(I) epitope, and A and B epitopes. Glycans with the moiety (Galβ1,4GlcNAc) are of type II. Further sugar additions to the branches of type II structures result in mature glycans with characteristic epitopes such as Lewis x, Lewis y, H(II) epitope, and A and B epitopes. The glycan maturation processing for type I and type II structures is summarized in Figure 4. Model predicted glycan abundances from MALDI TOF are listed together with reported literature data on the alteration of glycan processing in prostate cancer, especially trends in N-glycosylation for LNCaP cells. Literature values reported as: **1** LNCaP cells (mostly from PSA) unless indicated; **2** Prostate cancer tissue; **3** Seminal fluid; **4** Serum; **5** Other cell lines; **6** Metastases from human prostatic carcinoma; **A** Absent.

Predictions of the abundances of glycans based on the model analysis of the complete mass spectral data is shown in terms of types of glycans in Figure 5 and glycan moieties associated into blood group categories in Figure 6. The mass spectra for both high and low passage prostate cancer LNCaP cells are most abundant in high mannose glycoforms (Figure 5). Indeed, prostate-specific membrane antigen (PMSA) protein derived from LNCaP cells was found to contain high mannose structures [23] which indicates the generation of these structures from this cell line. In addition, a series of complex glycans with tetraantennary structures being the most abundant followed by biantennary and triantennary glycans are observed in both low and high passage cell lines (Figure 5) and were reported in [24]. Here we see that the more metastatic high passage cells have higher levels of hybrid glycans and lower levels of complex glycans, especially tetraantennary. Nearly 70%–80% of the hybrid and complex glycan structures (% based on hybrid and complex not total glycans) in both low and high passage LNCaP cells are core fucosylated (Figure 5). Indeed, previous studies have reported core fucosylated glycan structures as characteristic of PSA from LNCaP cells [8], [10], [25]. Complex glycans are mostly identified as non sialic acid capped complexes of the bi, tri and tetraantennary type. However, limited levels of monosialylated, bisected glycans structures and lactosamine repeats (Galβ1–4GlcNAc) are predicted. Glycan moieties of type II are predominant in both cell lines (Figure 5 and Figure 4). Note that a single glycan can contain more than one of a particular glycan moiety, so the abundances of some moieties can exceed 100% of the total number of glycans, used as the basis for this percentage. In terms of blood group structures which include antigens A, B, Le^a^, Le^b,^ Le^y^, Le^x^ and H in Figure 4, the H(II) and the Ley epitopes, containing α1,2-fucose linkages, are predicted in these types of prostate cancer cells, with greater abundance in high passage LNCaP cells (Figure 5). Indeed, these epitopes have been reported as characteristic markers for prostate cancer [7], [10], [26], [27], [28], [29], [30].

### Identification of LNCaP cell model characteristic enzymatic profiles from MALDI TOF

The concentrations of the glycan-processing enzymes were adjusted in the computational model until satisfactory agreement was obtained between the in silico mass spectral profile with the experimental mass spectral profile for both data sets. Shown in Table 1 (columns 4 and 5) are the adjusted enzyme activities that provided the best fit with both experimental mass spectral data sets and produced the glycan abundances in Figure 5 and 6. A comparison of the observed changes in the enzyme levels between high and low passage LNCaP cells provides a succinct way of interpreting the differences in glycan structural profiles between these LNCaP cells lines. In general we observe that model predicted enzyme activities are in qualitative agreement with available published enzyme values in terms of increasing and decreasing levels between cases. A description of some of the trends that were observed in the enzymatic activities corresponding to the resulting glycan profiles for the two LNCaP cell lines are described in following sections.

**Table 1:** Comparison of enzyme activities based on matching model to mass spectra along with gene expression data from the CFG Glycogene microarray version 3.

			Model-derived enzyme activities, min^−1^		Microarray gene expression data			
Enzyme	Enzyme name	EC number	Low passage LNCaP cells	High passage LNCaP cells	Probe ID	Gene ID	Low passage LNCaP cells	High passage LNCaP cells
ManI	α2-mannosidase I	3.2.1.113	3.41	4.33	NA			
ManII	α3/6-mannosidase II	3.2.1.114	2.93	2.24	180	MAN2A1	51.9	34
					181	MAN2A2	63	49.3
a6FucT	α6-Fuc-transferase	2.4.1.68	2.07	1.5	223	FUT8	31.6	37.2
					224	FUT8	28.4	25.4
GnTI	β2-GlcNAc-transferase I	2.4.1.101	0.86	0.72	294	MGAT1	86	104.5
GnTII	β2-GlcNAc-transferase II	2.4.1.143	3.86	3.23	293	MGAT2	258.7	233.4
GnTIII	β4-GlcNAc-transferase III	2.4.1.144	0.01	0	303	MGAT3	**2.6**	**2.4**
GnTIV	β4-GlcNAc-transferase IV	2.4.1.145	0.64	0.49	308	MGAT4A	2	2.8
					309	MGAT4B	217.9	148.3
					310	MGAT4B	413.3	322.7
					311	MGAT4B	240.6	192.4
GnTV	β6-GlcNAc-transferase V	2.4.1.155	0.97	0.79	292	MGAT5	**1.1**	**1.1**
					320	MGAT5B	29.3	**17.43**
iGnT	Blood group i β3-GlcNAc-transferase	2.4.1.149	0.01	0.01	312	B3GNT1	188.4	151.6
					296	B3GNT2	15.3	11
					297	B3GNT3	**2.5**	**2.9**
					298	B3GNT4	27.97	27.43
b4GalT	β4-Gal-transferase	2.4.1.38	8.97	9.18	267	B4GALT1	356.2	290.3
					268	B4GALT2	122.7	160.4
					269	B4GALT3	172.2	199.4
					272	B4GALT5	130.07	102.43
a3SiaT	α3-Sialyltransferase	2.4.99.6	0.002	0.002	385	ST3GAL3	38.9	52.1
					386	ST3GAL4	90.37	57.9
					388	ST3GAL6	**0.87**	**0.9**
IGnT	Blood group I β6-GlcNAc-transferase	2.4.1.150	1.18	1.62	313	GCNT2	**1.9**	**0.9**
					314	GCNT2	77.8	66.3
a6SiaT	α6-sialyltransferase	2.4.99.1	0	0	389	ST6GAL1	**4.1**	9
b3GalT	β3-Gal-transferase		0.46	0.41	262	B3GALT1	**1.9**	**3.3**
					263	B3GALT2	**0.3**	**0.2**
					265	B3GALT5	**2.6**	**3**
FucTLe	α3/4- Fuc-transferase III	2.4.1.65	0	0	213	FUT3	14.2	26.8
a3FucT	α3- Fuc-transferase	2.4.1.152	0.03	0.04	215	FUT5	**6.2**	**6**
					216	FUT6	**26.5**	**28.8**
					217	FUT6	**8.6**	10.3
					218	FUT6	**3.4**	**3.1**
					219	FUT6	**0.5**	**1.4**
					220	FUT6	**2.8**	**5.7**
					221	FUT6	17.8	**23.9**
					214	FUT4	9.2	9.9
					222	FUT7	**3.5**	**1.9**
					225	FUT9	**0.2**	**0.5**
FucTH	α2- Fuc-transferase, Se, H	2.4.1.69	0.02	0.06	207	FUT1	43.3	74.4
					211	FUT2	**5.5**	**3.7**
					212	FUT2	**6.4**	**1.4**
GalNAcT-A	Blood group A α3-GalNAc-transferase	2.4.1.40	0.001	0	284	ABO	**1.6**	**1.3**
GalT-B	Blood group B α3-Gal-transferase	2.4.1.37	0	0	284	ABO	**1.6**	**1.3**

Experimental gene expression results are shown for low and high passage LNCaP human prostate cancer cells (CFG ID: MAEXP_291_040606). Bold entries indicate microarray results below the reported significance level.

#### α2-Fuc-transferase (FucTH) is elevated in high passage, androgen independent LNCaP cells

The highest percentage change in enzyme levels the model predicts for the high passage LNCaP cells with respect to the low passage cells is an increase in α1,2-fucosyltransferase (FucTH) activity (see Table 1 columns 4 and 5). As shown in the processing diagram of Figure 4; FucTH mediates α1,2 linkages of fucose to a terminal galactose moiety of Type II (Galβ1,4GlcNAc) or Type I (Galβ1,3GlcNAc) structures (Figure 4), and it is associated with expression of blood group H (Figures 4 and 6). The H epitope can be further augmented by other fucoses to form Lewis b (Le^b^), and Lewis y (Le^y^) antigens (Figures 4 and 6). Indeed, FucTH enzymatic activity has been reported in LNCaP cells. For example Chandrasekaran et al. purified FucTH from LNCaP cells and characterized several substrates for this enzyme [35]. Furthermore, Marker et al. proposed that α1,2 fucose activity in LNCaP cells may modulate pathological prostatic growth [28]. These previous findings are consistent with the presence of FucTH activity in LNCaP cells predicted by the model.

#### Sialic acid content in LNCaP cells

The model simulation based on the mass spec profile predicted low levels of sialylated glycan structures (Figure 6) in both LNCaP cell lines, which correspond to minimal levels of α3-Sialyltransferase (a3SiaT) (Table 1-columns 4 and 5). In previous studies, glycan sequencing of released glycans using high performance liquid chromatography (HPLC) coupled with exoglycosidase digestions on PSA from LNCaP cells at passage 70–75 showed only neutral (nonsialylated) structures [10]. In another glycosylation study of the same tumor cell line, researchers also noted the lack of sialic acid on glycan structures of PSA using lectin analysis coupled with glycan sequencing [25]. However, it should be noted that some other studies did report sialic acid present on LNCaP cells [8] [33]; the differences in these reports may be due to clonal variability, number of passages, analytical method sensitivity, and general cell culture conditions.

#### Other enzymatic trends

Additional enzymatic trends predicted by the model are the presence of b4GalT enzyme that catalyzes Type II (Galβ1,4GlcNAc) glycans and, in comparison, low levels of the b3GalT enzyme that catalyzes Type I glycans (Galβ1,3GlcNAc) (Table 1 columns 4 and 5). Model capability to differentiate type I and type II glycans is based on the presence of iGnT and IGnT enzyme activities as shown in Figure 4 that act on type II glycans but not on type I glycans. Interestingly, the interpretation of the mass spectral data thus points to the predominance of type II glycans and their derivative products in comparison to Type I glycans abundance in these LNCaP cell lines (Figure 6).

Previous studies of PSA from LNCaP cells noted the presence of Type II structures using *Erithrina cristagalli* lectin (ECL) [25], and the presence of the H(II) epitope (Fucα1,2Galβ1,4GlcNAc) [10], [28]. Indeed, the model outputs for both LNCaP cell lines confirms the presence of the enzymes involved in the processing of the Type II glycans moieties, which include LacNAc (Galβ1,4GlcNAc), Le^x^ (Fucα1,3Galβ1,4GlcNAc), H (II) epitope (Fucα1,2Galβ1,4GlcNAc), and Le^y^-(Fucα1,3(Fucα1,2Galβ1,4)GlcNAc) structures (Figure 6 and Figure 4). Alternatively, the lack of type I based Lewis a (Le^a^) and Lewis b (Le^b^) antigens has been reported in prostatic carcinoma [34] and is in agreement with model predictions for the absence of Le^a^ and Le^b^ epitopes (Figure 4 and 6). Correspondingly, the model predictions show a lack of FucTLe activity in Table 1, which catalyzes the α-4 fucose addition for generating Le^a^ and Le^b^. The model also predicted the presence of core Fucose glycans arising from α6-Fucosyltransferase, which agrees with studies on PSA from LNCaP indicating the presence of glycans with α6-core Fucose [10]. Consistent with model predictions showing Blood group A α3-GalNAc-transferase (GalNacT-A) and Blood group B α3-Gal-transferase B (GalT-B) enzymes having low activity (Table 1 columns 4 and 5 and Figure 4), minimal or lack of A and B blood group antigens has been reported on prostate cancer tissues and observed in the model [26], [27] (Figure 6). Although no information has been reported on GnTIII, in LNCaP cells, this enzyme is predicted to be low or marginal in the model simulations (Table 1 columns 4 and 5).

### Incorporating gene expression data

Mapping of model enzymes Table 1 (column 1) to gene probes (columns 6 and 7) on the Consortium of Functional Glycomics (CFG) Glycogene microarray (Glycochip version 3, CFG) was performed for both low and high passage human prostate LNCaP cell line data available at the Consortium of Functional Glycomics website (MAEXP_291_040606) and also included in Dataset S3 [22]. Listed in columns 8 and 9 of Table 1 are the observed changes in expression levels of these genes as determined from mRNA analysis of microarrays for the low passage and high passage LNCaP cells. These expression signals were obtained by averaging three replicate samples for each glycogene in the microarray and represent the average relative abundance of a transcript. The glycogenes were assigned calls by CFG of present (P), marginal (M) or absent (A) (more information on the classification criteria is found in Materials and Methods). In Table 1 the marginal or absent calls are indicated with numbers in bold. Note that more than one gene can encode the same type of enzyme activity.

In addition to the genes for N-Glycan processing as discussed for Table 1, the glycochip version 3 includes genes for many other glycosylation-related genes. However, in some cases not all the genes that encode for a given enzyme are included on the CFG version 3 microarray. Interestingly, the largest shifts in gene expression observed between the two types of prostate cancer cell types from the glycochip are those that encode for the enzyme glucuronosyltransferase (EC 2.4.1.17), which is involved in androgen/estrogen metabolism but has no effect on N-glycan structure [36], [37], [38].

### Comparison of experimental glycosyltransferase gene expression levels to model-derived enzymatic activity levels obtained from MALDI TOF data

In general transcript expression levels from the microarray are consistent with the enzyme activities resulting from the model, at least in terms of the presence or absence of enzymes in the model and on the gene expression array. Enzymes whose genes are classified as marginal or absent in the microarray data include GnTIII, a6SiaT, GalTB and GalNacT-A (Table 1 columns 8 and 9 in bold). Interestingly, enzymatic activity levels for this same collection of enzymes were also predicted by the model to be low or zero independently based on fits of the mass spectral data (Table 1 columns 4 and 5).

The most significant percentage shift in enzyme activity that differentiates high and low passage LNCaP cells was increased FucTH activity in high passage or androgen independent LNCaP cells. Although the model based solely on mass spectrometry measurements deduced this finding of increased FucTH activity, the uptick in FucTH expression was also correlated with mRNA microarray expression data for the FUT1 gene (Table 1 columns 8 and 9). The FUT1 gene has been experimentally identified in LNCaP cells and prostate cancer tissues as responsible for production of the H (II) epitope [10], [28].

In addition model-fitted mass spectra data indicate a lower capacity for generating type I glycans relative to type II glycans as b3GalT activity is lower than b4GalT activity in Table 1. This finding also correlated with the relative gene expression data in that the expression of b3GalT genes encoded by B3GALT1, B3GALT2, and B3GALT5 are marginal in both data sets from both cell lines (Table 1 columns 8 and 9). In contrast, gene expression levels for the B4GALT1, B4GALT2, B4GALT3 and B4GALT5 (Table 1 columns 8 and 9) encoding b4GalT activity for type II glycans is robust in both cell lines as it is the generation of type II glycans as indicated by the model fitting of mass spectral data and predicted b4GalT enzymatic activity.

While there were many consistencies between model interpretation of mass spectrometry and gene expression profiles, there were also some disagreements between the model-calculated enzyme levels and the expression levels obtained from mRNA, such as for GnTV, a3SialT and FucTLe. For some enzymes, these differences can be attributed to shortcomings in the mRNA profiling chips. A case in point is that the inactivity of a probe set for MGAT5 (one of two genes encoding GnTV activity) in the glycochip version 3 from CFG led to a reported lack of expression of this gene despite the presence of GnTV activity in the results modeled from the mass spectral data (Table 1 column 4 and 5). Interestingly, by reviewing all experimental cases run with the glycochip version 3 for all cell lines posted on the CFG we found that the probe set for MGAT5 gene was inactive. Furthermore, the presence of GnTV indicated by the model interpretations of the mass spectral data agrees with a previous experimental study that reports positive GnTV enzymatic activity using a zymography assay and active expression of MGAT5 through RT-PCR in LNCaP cell lines [32]. Results from model testing of this proposed deficiency in the glycochip version 3 are discussed in Text S2, Figure S2, and Figure S3. These findings support the interpretation that the inactivity of the GnTV in the microarray data is due to a defect in this specific probe on the microarray.

For other enzymes, the lack of model agreement with gene expression data is likely due to the scope of the current model. For example, the microarray data indicates significant levels of α3SiaT mRNA for both LNCaP cell passages, although the enzymatic model interpretation from MALDI TOF indicated that LNCaP cells have low a3SialT activity. Interestingly, analysis of expression of genes associated with sialic acid biosynthesis, a feature not included in the current model, indicates that the transcript levels for the GNE gene (that encodes the bifunctional UDP-N-acetylglucosamine 2-epimerase/N-acetylmannosamine kinase) for both cell lines are interpreted as absent. Bifunctional GNE catalyzes two critical steps involving sequential reactions in the biosynthesis of the sugar nucleotide CMP-Neu5Ac (CMP-sialic acid) (Figure S4, Text S3), which is the co-substrate for a3SiaT. The α2,3- or α2,6-linked sialylated N-glycans are generated by the transfer of the sialic acid (Neu5Ac) group from the nucleotide donor sugar CMP-Neu5Ac onto the oligosaccharide acceptor ending in a galactose (Gal) residue. Thus, the formation of a limited number of sialylated glycoforms may be due to low levels of the nucleotide sugar substrate, CMP-Neu5Ac, rather than due to a limitation in the a3SiaT activity as currently manifested in the model. While the current model has focused on glycosyltransferase activities, it can be readily expanded to include other metabolic reactions such as the generation of sugar nucleotides including CMP-Neu5Ac and others.

For the FucTLe enzyme, the model predictions show lack of activity, while the microarray data shows gene expression signal for FucTLe. Both FucTLe and a3FucT enzymes catalyze the addition of α-3 fucose to GlcNAc residues on type II chains as shown in Figure 4. They only differ on Type I chains, in the generation of Le^a^ and Le^b^ structures. The lack of these Le structures makes it difficult for the model to separate the two enzyme activities and at least part of the activity the model shows for a3FucT may be due to FucTLe.

### Glycan model profiling using enzymatic transcript expression data

As an alternative predictive approach, the potential also exists for using gene expression profiles to estimate changes in enzyme activities. Appreciating that mRNA levels do not always reflect enzyme levels directly, we assumed shifts in gene expression data were related to shifts in enzyme levels. These shifts in gene expression were then used to predict the corresponding shifts in glycan profile and abundances for high and low passage LNCaP cells. To get the transcript expression level of the enzymes in the model, the average of all mRNA-microarray gene signals corresponding to each enzyme was used. Next the ratios of average signals (high/low passages) for each enzyme were used as inputs to the N-glycosylation model based on the assumption that they are estimates of the relative enzyme levels for high and low passage LNCaP cells. The implementation of this methodology into the model also required adjusting the enzyme levels in the model to match the experimental mass spectra data for one case (low passage for this study) while maintaining the enzyme activity ratios to be equal to the experimental values obtained from the microarray data (Table S1). The ratios of expression levels of high to low passage cells were then used to predict the enzyme levels for the high passage case and the resulting glycan profile predicted. No constraint was placed on the concentrations of GnTV or ManI in the model as these enzymes have either a probe defect (GnTV) or are missing in the glycochip version 3 (Man I).

**Figure 7:**
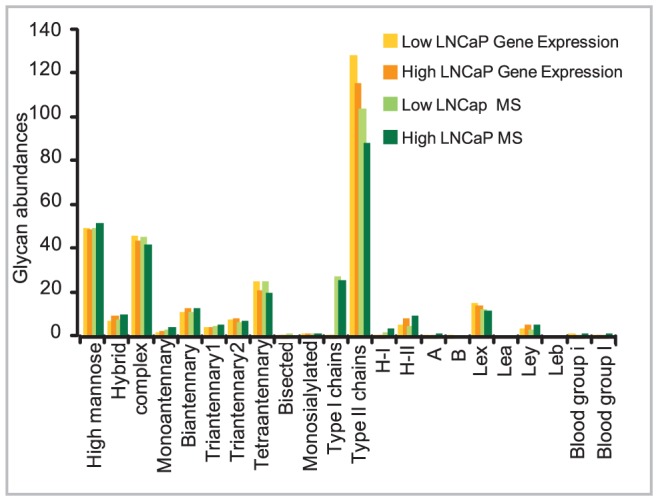
Comparison of model predicted glycan abundances (%) from MALDI TOF MS and fixed Gene Expression ratios for low passage, androgen dependent and high passage, androgen independent LNCaP cells. Percents values can be more than 100% as the number of structures counted within the glycans can overpass the number of glycans.

The results of keeping the enzyme ratios constant and equal to the microarray ratios are shown in Figure 7, which show the abundances of different categories of glycans. The percentage of different structures predicted by the model using MALDI-TOF mass spectral data alone (green bars) are compared to the abundances obtained after fixing the gene expression mRNA ratios (orange bars). Predictions for the low passage, androgen dependent LNCaP cells are indicated by light green bars for mass spectral data and light orange bars for mRNA data. Similarly, predictions for high passage, androgen independent LNCaP cells are indicated by dark green bars for mass spectra data and dark orange bars for mRNA data. This approach allows the model to predict changes in glycan structure profile and glycan abundances based on comparative N- glycosylation enzyme gene expression data.

Although constraining model fitting to fixed gene expression ratios resulted in a higher RMS error (lower model agreement) with respect to the model fitting to the mass spectra data alone (as would be expected for additional model constraints), the trends in glycan structures and abundances are comparable in both cases with a few minor exceptions. In general, glycan structure prediction from both data sets (gene expression and mass spectra vs. mass spectra alone) show consistency in terms of presence, absence and increased or decreased abundances of glycans in high passage with respect to low passage LNCaP cells. For example, both model predictions with mass spectra and gene expression data suggest an increase in abundance of hybrid and biantennary structures in the high passage cells and a corresponding decrease in the tetraantennary structures. Both mRNA data and models predict high levels of type II chains in both low and high passage LNCaP cells, reaffirming that these cell lines contain predominantly type II glycans. The model based on gene expression data suggested lack of type I glycans, principally due to imposing a strict restriction on b3GalT which in reality may not be as strict as the mRNA data for the genes encoding this enzyme are classified as either marginal or absent.

## Discussion

In this study, a systems biology computational model that connects diverse experimental data sets was used to evaluate N-glycan data from MALDI-TOF mass spectra and mRNA expression arrays for androgen independent, high passage LNCaP cells, and androgen dependent, low passage, LNCaP cells. Most significantly, insights into the N-glycosylation processing for LNCaP high and low passage cells were found based on model predictions of enzyme activities, glycan structures and gene expression profiles. The model was also useful for identifying consistencies as well as incongruities between glycan structural information and gene expression data.

The N-glycosylation model identified and quantified glycan structural details not typically derived from single-stage mass spectral or gene expression data, such as the type of fucosylation (Fuc-α1,2-Gal vs. Fuc-α1,3-GlcNAc and Fuc-α1,6-GlcNAc), the predominance of Type II chains (Gal-β1,4-GlcNAc) versus Type I chains (Gal-β1,3-GlcNAc) or the number of antennae. This is possible by analyzing the total mass spectrum in terms of the underlying processing events and enzyme activities that generate both the individual structures and the assemblage of structures resulting in the complete mass spectrum. For example, Fuc-α1,2-Gal can be differentiated from Fuc-α1,3-GlcNAc because they include the different molecular weight linked sugars of Gal and GlcNAc. More relevant to the current modeling approach, Fuc-α1,3-GlcNAc and Fuc-α1,6-GlcNAc can be differentiated because, even though both include the same molecular weight linkages, Fuc-α1,6-GlcNAc is added to the glycan core early in processing, while the Fuc-α1,3 is only added later to GlcNAc on one of the N-glycan branch extensions. The presence of a collection of mass spec peaks provides a fingerprint that the model can interpret to indicate whether this fucose is added earlier (Fuc-α1,6-GlcNAc) or later (Fuc-α1,3-GlcNAc) in the N-glycan processing pathway. Thus the model creates a picture of the complete N-glycan processing, including enzyme activity levels acting sequentially through the secretory apparatus that is consistent with the entire collection of glycan peaks across the molecular weight spectrum.

Comparison of the underlying enzyme activities derived by the model from the mass spectral data with changes in gene expression levels measured by the CFG glycogene microarray show them to be consistent for most enzymes, which tends to verify that the model-derived shifts are meaningful. The agreement also suggests that the model could be used to predict shifts in glycan structure from a well-defined base case based on changes in microarray expression data when MS data for other cases is not available.

It is important to note that aberrant N- and O-glycosylation are important features of cancer cells. Indeed a number of the genes included in the model act on both N-glycans and O-glycans, while the model infers changes in the total enzyme activities based only on the observed shifts in N-glycan structures. Thus if the levels of O-glycan structures that compete with N-glycan structures for a number of enzymes changes significantly, the fraction of those enzymes activities that are available for N-glycan processing could also change, distorting the comparison between model predicted enzyme activities and enzyme gene expression levels. While our N-glycosylation model gave very good agreement between the model-predicted and measured mass spectra, it is expected that the incorporation of O-glycosylation together with N-glycosylation will improve the model predictability. In addition, these competing reactions will be better modulated in the model. Implementation of O-glycosylation in the current model framework is possible since kinetic parameters for the corresponding O-glycan enzymes as well as experimental data are available to tune the model. Of course, processing larger data sets including O-glycans may very well elucidate limitations in the model that will need to be addressed through appropriate modification of model parameters.

Interestingly, the most significant difference found between high and low passage prostate cancer cell lines was the increase in expression of α1,2-Fuc-transferase (FucTH) enzyme in the high passage LNCaP cells, as predicted by the model based on mass spectrometry and verified by the gene expression data. The microarray data indicates that the FUT1 gene is predominant in high passage LNCaP cells with respect to low passage LNCaP cells. The FUT1 gene has been experimentally identified in LNCaP cells and prostate cancer tissues and associated to the H (II) epitope (Fucα1,2Galβ1–4GlcNAc) [28]. Moreover, the presence of Fucα1-2Gal residues that results from the enzymatic action of FuTH has been reported in PSA from LNCaP cells [10], [28], [31], [35].

The high passage LNCaP cells in this work were obtained from low passage LNCaP cells (androgen dependent) after successive passages and they have diverged into an androgen independent state. Our finding that high passage LNCaP prostate cancer cells (androgen independent) have increased levels of the enzyme FucTH (FUT1 gene) responsible for α1, 2-fucosylation and the H (II) and Le^y^ epitopes with respect to low passage LNCaP cells may represent a potential marker of higher malignancy or androgen refractory prostate cancer cells and may be relevant in diagnosing prostate cancer stage. For example it may be possible to compare glycan mass spectra of PSA concentrated from blood serum, presumably originating in cancer cells, to PSA from semen samples, derived mostly from normal prostate cells, to evaluate the stage of the cancer.

Our results also indicate that b4GalT is present in both high and low passage LNCaP cells. The most expressed member of the family is the B4GALT1 gene followed by the B4GALT3 gene. Evidence of b4GalT in prostatic cancer samples is found by detection of Galβ1,4GlcNAc (Type II) structure with the *Erithrina cristagalli* lectin (ECL) in PSA from prostate cancer serum and PSA from LNCaP medium as compared to seminal plasma (normal control) [25]. This type II structure was also detected with a set of lectin-immobilized columns together with enzyme-linked immunosorbent assays (ELISA) on prostate cancer serum PSA and LNCaP cells PSA as compared to benign prostate hyperplasia (BHP) serum PSA [31].

Interestingly, screened experimental data on prostate cancer predominantly reports the presence of Galβ1,4GlcNAc (Type II) glycans and some of its derivatives and almost no information is found for Galβ1,3GlcNAc (type I) glycans. In agreement with that, the model predicted the presence of H type II (Fuc1,2Galβ1,4GlcNAc) and Le^y^-(Fuc1,2Galβ1,4GlcNAc Fuc1-3) glycans and also increased levels of these epitopes in the more metastatic high passage LNCaP cells. Importantly, several previous studies have reported type II based epitopes H [10], [26], [27], [28] and Le^y^ as blood group antigens as characteristic of prostate cancer [7], [29], [30], [39]. For example, lectin histochemistry comparisons between normal human prostate and prostatic carcinoma tissues show increased expression of galactose (using DSA lectin suggesting presence of Galβ1,4GlcNAc), and fucose [6] (using UEA-I, a marker for the H antigen). Also, investigations of PSA serum from 40 patients revealed an increase in the glycans containing Fucα1,2Galβ1,4GlcNAc and GalNAcβ1,4GlcNAc for patients with prostate cancer as opposed to those with benign prostatic hyperplasia (BPH) [31]. Moreover the production of the H (II) epitope (Fuc1,2Galβ1–4GlcNAc) has been associated with the potential for carcinogenic cell proliferation (Marker et al, 2001). Most importantly, these findings reflect trends predicted by both gene expression data and mass spectral data.

A corollary glycan signature predicted by the model from mass spectra is lower relative levels of the b3GalT enzyme, which was even more pronounced in the gene expression data as indicated by the low levels of transcript signals for the genes encoding for b3GalT. In addition to the lower abundance of type I glycans in both cell lines, derivatives including Le^a^ and Le^b^ epitopes were also absent. These observations are in agreement with studies reporting low levels or the complete absence of type I based antigens Le^a^ and Le^b^ [34] in prostatic carcinoma. Additionally, the A and B blood group antigens from type I and Type II glycans, were predicted absent or minimal in agreement with [26], [27].

Moreover, the comparisons of model predictions from glycan structural data with gene expression findings pointed to deficiencies in the mRNA microarray, such as a lack of a sensitive probe for the MGAT5 gene for GnTV. This was further confirmed by computationally suppressing GnTV enzyme activity and demonstrating that the modified model could not regenerate the experimental mass spectra (Text S2, Figures S2 and S3). Indeed, the presence of GnTV indicated by the computational model agrees with a previous experimental study that reports GnTV enzymatic activity using zymography assay together with detection of expressed MGAT5 using RT-PCR in LNCaP cell lines [32].

In summary, this study demonstrates the potential of systems glycobiology approaches as means to connect and interpret disparate data sets obtained with widely different experimental methods, in this case mass spectral data and gene expression profiles. The resulting model approach allows users to better understand N-glycosylation processing events in a prostate carcinoma cell line and also helps to define consistent patterns and incongruities between data obtained from mass spectrometry and microarrays. Consistent patterns observed in data sets from multiple methods may represent potential glycan biomarkers for cancer and other diseases. Furthermore, by using these computational tools, we have been able to show how changes in the mRNA data can be used to describe glycan patterns in low and high passage LNCaP cells. In this way, the model can increase the value of current mRNA profiling data as a useful tool for indicating changes in glycan processing. This effort will contribute significantly to the current need for bioinformatics and systems biology tools in glycobiology. The method allows users to interpret, integrate and compare multiple complex data sets in order to identify and validate critical biomarkers involving N-glycosylation processing in normal and diseased cells and tissues.

## Materials and Methods

### Model validation

The model has been extensively validated with several public available mass spectra data sets (http://www.functionalglycomics.org) of mammalian human and CHO cells. Methods demonstrated in previous publications of the model (Krambeck 2005 [20], and Krambeck 2009 [19]) with other experimental data sets enable using the model comparatively between a control case and other case/s. These methods have the advantage of allowing a common adjustment to the model for two or more cases while using only the enzyme concentration levels to differentiate the samples. This principle limits case-to-case variations as much as possible to just the enzyme concentrations while holding almost all other model parameters to uniform values for all cases. In this respect the accuracy of this approach depends on how sensitive the predictions are to the assumed values of unknown parameters in the model. For example sensitivity analysis on the effect of total glycan concentration on the enzyme levels shows that the predicted effects of changes in enzyme concentration drive similar shifts in glycan profiles for different total glycan concentrations. Similar studies have been applied to analyze sensitivity effects to assumed values of other parameters. As the range of experimental data the model can accommodate expands, the more robust and reasonable the model results will become. Thus establishing a wide database of analyzed glycans from numerous cells and tissues is essential to improving model robustness.

### Reaction network generation

**Figure 8:**
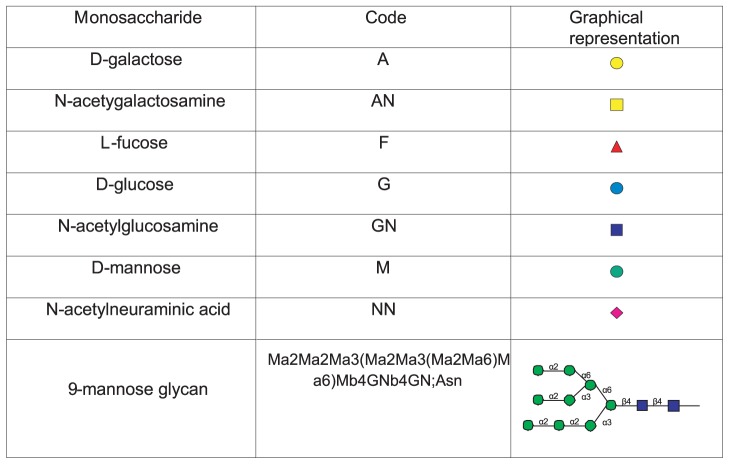
Sugar codes used in formulas and example of a glycan structure using condensed formulas.

In the current model framework, glycan structures are expressed using a condensed version of IUPAC linear formulas [40] with some minor modifications. The first modification is to order the branches at a branch point based just on the branch locants (the carbon atom numbers of the attachment points of each branch) without regard to the lengths of the branches as is used by the IUPAC scheme. In addition the sugar abbreviations have been replaced with the shorter abbreviations of the Linear Code [41], but we have not used the complicated branch ordering scheme of the Linear Code. Figure 8 shows the sugar symbols used in glycan structures for our model as well as an example of the condensed linear formulas used to represent glycan structures for a 9 -mannose glycan. This scheme provides linear formulas that are general, are easily readable by humans, are unique for each glycan structure, and allows the model to apply to N-glycans, O-glycans and glycolipids.

### Enzyme reaction rules

Table 2 gives a list of the enzymes included in the current model and the set of reaction rules for each enzyme. These are sufficient to produce most of the N-glycans present in human cells. The basic idea is that the “Substrate” column is a substring of the linear formula that must be present for the enzyme to act. The “Product” column specifies what the substrate string is replaced with through action of the enzyme. The “Constraint” column specifies a set of additional conditions that must be satisfied for the enzyme to act. These conditions are usually the presence or absence of another substring somewhere in the substrate formula. These are combined using the “not” operator (∼), the “and” operator (&) and the “or” operator (or), with parentheses as appropriate. To simplify these expressions a number of additional conventions have been added. All substrate formulas are assumed to be enclosed in parentheses before searching for the substrate substring. Thus an initial “(” always indicates the terminal end of a branch. Other codes and abbreviations used in formulating the reaction rules are summarized in Table 3. (See example in Table S2 and further detail in Text S1).

**Table 2:** Current reaction rules.

Index	Enzyme	Substrate	Product	Constraint
1	ManI	(Ma2Ma	(Ma	∼*2Ma3(…Ma6)Ma6 & ∼Ga3
2	ManI	(Ma3(Ma2Ma3(Ma6)Ma6)	(Ma3(Ma3(Ma6)Ma6)	∼Ga3
5	ManII	(Ma3(Ma6)Ma6	(Ma6Ma6	(GNb2|Ma3 & ∼Gnbis
6	ManII	(Ma6Ma6	(Ma6	(GNb2|Ma3 & ∼Gnbis
7	a6FucT	GNb4GN	GNb4(Fa6)GN	GNb2|Ma3 & #A = 0 & ∼Gnbis
8	GnTI	(Ma3(Ma3(Ma6)Ma6)Mb4	(GNb2Ma3(Ma3(Ma6)Ma6)Mb4	
9	GnTII	(GNb2|Ma3(Ma6)Mb4	(GNb2|Ma3(GNb2Ma6)Mb4	
10	GnTIII	GNb2|Ma3	GNb2|Ma3(GNb4)	∼Ab & ∼Gnbis
11	GnTIV	(GNb2Ma3	(GNb2(GNb4)Ma3	∼Gnbis
12	GnTV	(GNb2Ma6	(GNb2(GNb6)Ma6	∼Gnbis
13	iGnT	(Ab4GN	(GNb3Ab4GN	∼*_Ma3|Mb4
14	b4GalT	(GN	(Ab4GN	∼*GNb4)(…Ma6)Mb4
15	a3SiaT	(Ab4GN	(NNa3Ab4GN	
16	IGnT	(Ab4GNb3Ab	(Ab4GNb3(GNb6)Ab	
17	a6SiaT	(Ab4GN	(NNa6Ab4GN	
18	b3GalT	(GN	(Ab3GN	∼*GNb4)(…Ma6)Mb4
20	FucTLe	Ab3GNb	Ab3(Fa4)GNb	
21	FucTLe	(…Ab4GNb	(Fa3(…Ab4)GNb	
22	FucTH	(Ab3GNb	(Fa2Ab3GNb	
23	FucTH	(Ab4GNb	(Fa2Ab4GNb	
24	a3FucT	(…Ab4GNb	(Fa3(…Ab4)GNb	
25	GalNAcT-A	(Fa2Ab	(Fa2(ANa3)Ab	
26	GalT-B	(Fa2Ab	(Fa2(Aa3)Ab	

**Table 3:** Codes used for reaction rules in Table 2.

Symbol	Meaning	String expression
…	Ligand	Any string (possibly empty) with all parentheses matched.
_	Continuation	Any string (possibly empty) where every “(” is matched with a following “)”
|	Possible branch point	Empty string or (…).
*	Reaction site	Position of first difference between product and substrate strings
Gnbis	Bisecting Gn	Ma3(GNb4)(…Ma6)Mb4
#	Number of	
∼	Logical not	
&	Logical and	
or	Logical or	

The model kinetic reaction network (Table S3) is generated by a series of string searches and substitutions which begin with a list of starting structures (9 and 8 -mannose glycans e.g. Figure 8). Each substrate rule and corresponding constraint rule is then applied to each structure in the list to determine which structures are substrates for each rule. For structures that satisfy the rules, the product structure is determined, essentially by substituting the product substring of Table 2 for the substrate substring, taking the various abbreviations into account. If the structure is not already in the list of structures it is added to the list. At the same time, the new reaction is added to a reaction list. The reaction list includes the enzyme, substrate, cosubstrate, product and coproduct strings for each reaction. This process is repeated until no new reactions can be generated. A molecular mass cutoff to limit the size of the glycans generated to those observable in the mass spectral data is included. To reduce the size of the model further a network pruning method was used based on roughly estimating the abundances of the structures and dropping those structures of negligible abundance. Starting with the 9-mannose glycan shown in Figure 8, and including an inert structure with an additional glucose residue (Ga3), the rules of Table 2 generate a reaction network containing 10,809 structures and 28,797 reactions. The maximum mass cutoff used to generate this network was 4000 on a permethylated basis and network pruning was enabled.

### Reaction kinetics

Consider the glycosylation of a glycan Pi with a monosaccharide S catalyzed by an enzyme E. Assuming that the donor cosubstrate is UDP-S, (µM) the overall reaction is shown in equation (1):
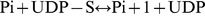
(1)


Assuming that the product Pi+1 competes for the same enzyme site as the substrate Pi (µM), that the donor cosubstrate UDP-S occupies a second site on the enzyme, and that the reaction is reversible, the Michaelis-Menten kinetic equation takes the form shown in equation (2).
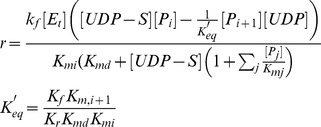
(2)


Here [E_t_] is the Enzyme concentration, µM, k_f_ (min^−1)^ and k_r_ (min^−1^ µM^−1)^ are the forward and reverse rate coefficients, K_mi_ and K_md_ are the dissociation constants for the substrate and donor cosubstrate in µM, and 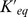 is the apparent equilibrium constant for the overall reaction. The symbols 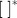 in equation (2) denote equilibrium concentrations. The subscript “j” in the summation in the denominator is taken over all the substrates that compete for the same enzyme. A derivation for equation (2) is given in the KB2005 model [20].

### Kinetic parameters and adjustment rules

The values of the kinetic parameters k_f_, K_m_ and K_md_ for a given enzyme can vary significantly for different substrates. This was accommodated by selecting base values for these parameters for each reaction rule and adding a set of structure-dependent adjustment rules. Development of these parameter values and adjustments for CHO and human cell enzymes are detailed in [19], [20]. The base parameter values currently used for each of the reaction rules in Table 2 are shown in Table 4. Adjustment rules for the parameters are given in Table 5. Each adjustment rule includes a condition on the substrate structure and multipliers to apply to each of the three parameters whenever the condition is satisfied.

**Table 4:** Base reaction rate parameters corresponding to the rule indices in Table 2.

Index	Enzyme	kf	Km	Kmd
1	ManI	1923.75	827	0
2	ManI	1923.75	5000	0
5	ManII	1923.75	200	0
6	ManII	1923.75	100	0
7	a6FucT	253	25	46
8	GnTI	990	260	170
9	GnTII	1320	190	960
10	GnTIII	607.2	190	3100
11	GnTIV	187	3400	8300
12	GnTV	1410	130	3500
13	iGnT	24.66	700	55
14	b4GalT	8712	150	0
15	a3SiaT	484.1	260	57
16	IGnT	25	440	0
17	a6SiaT	25	180	0
18	b3GalT	25	110	250
20	FucTLe	481	1900	10.5
21	FucTLe	25	1600	5
22	FucTH	28.2	1500	108
23	FucTH	28.2	5700	108
24	a3FucT	25	1400	9
25	GalNAcT-A	294	15	13
26	GalT-B	390	281	285

**Table 5:** Adjustment rules and factors corresponding to the rule indices in Table 2.

Adjust	Index	Rule	kf	Km	Kmd
1	1	#M = 9	1	182.052	1
2	1	#M = 8	1	4.21136	1
3	1	#M = 7	1	1.72953	1
4	1	#M = 6	1	1	1
5	10	∼GNb2|Ma6	1	20	1
6	11	∼GNb2|Ma6	1	5	1
7	11	Ab4GNb2|Ma6 or Ab4GNb6)Ma6	1	1.5	1
8	11	GNb6)Ma6	1	0.178	1
9	11	GNb4(Fa6)GN	1	1	1
10	12	GNb4)Ma3	1	0.69231	1
11	12	GNb4(Fa6)GN	1	1	1
12	13	*_Ma3	1	10	1
13	13	*_GNb2Ma6	1	4	1
14	13	*_GNb2Ma3	1	4	1
15	14	*_GNb6)Ma6	1	0.8	1
16	14	*_GNb2|Ma6	1	5.4	1
17	14	*_GNb4)Ma3	1	0.66667	1
18	14	*_GNb2|Ma3	1	1	1
19	14	Gnbis & GNb2|Ma6	1	3.62	1
20	14	∼GNb2|Ma6	1	26.6667	1
24	15	#NN>1	1	5	1
25	20	Fa2Ab3*	0.051975	0.10526	0
26	20	NNa3Ab3*	0.051975	0.35263	0
27	21	(*Fa2Ab4	1	0.6875	0
28	21	(*NNa3Ab4	1	0.0625	0
29	24	(*Fa2Ab4	4.08	0.5	1

### Simulation model

The Golgi compartments were modeled as well-mixed reactors with bulk flow of the contents from each compartment to the next compartment in line while the enzymes remain fixed in the compartments. At steady state the concentrations of the various structures satisfy the balance equations.
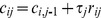
(3)where *c_ij_* is the concentration of structure *i* in compartment *j*, *τ_j_* is the residence time of compartment *j*, and *r_ij_* is the net rate of production of structure *i* per unit volume of the compartment due to all the biochemical reactions that occur. The total concentration of *N*-glycans, *c_tot_*, is the same in each compartment and is given by *pτ*_1_/*v*_1_, where *p* is the total production rate of glycans and *v*_1_ is the volume of compartment 1. In the balance equations for the first compartment the concentration *c_i_*_0_ is given by the fraction of total glycans that initially have structure *i* multiplied by *c_tot_*. Using the above Michaelis-Menten kinetics for the glycosylation reactions, equations were derived to solve for the concentrations of each of the individual glycan structures in each of the Golgi compartments.

### Numerical methods

The model equations are nonlinear algebraic equations which are solved for the concentrations of each of the structures in each of the four Golgi compartments. These are solved using a constrained Newton-Raphson method with the Harwell MA28 sparse linear solver (HSL 2002). The efficiency of a sparse linear solver for large numbers of variables depends on the problem Jacobian being sparse. The Michaelis-Menten denominator terms in Equation (2) involve a large number of species that compete for each of the enzymes. This could make the Jacobian matrix rather dense. To avoid this, the denominator terms for each enzyme are formulated as separate variables with equations added to specify how the denominators are calculated. This confines the equations with large numbers of variables to only one for each enzyme. Analytical derivatives were used. While each compartment could be solved separately in sequence to give four subproblems, each one fourth the size, this was not found to be necessary.

In addition to solving the model for a given set of model parameters, provision was also made to adjust parameters to match a given set of data. This was done using the Marquardt-Levenberg method with analytical derivatives [42]. The same method was used for optimizing model parameters to achieve a desired distribution of glycan structures.

The Marquardt-Levenberg method is typical of nonlinear optimization algorithms in that it makes use of a sequence of local linear approximations to the nonlinear model to converge to a solution that is a local optimum. Except in special cases there is no way to determine whether the nonlinear problem possesses an even better solution far removed from this point. Experience in using this method shows, however, that if a reasonably good fit is obtained with the local optimum it is also a global optimum for the parameter estimation problem. Robust solution methods were devised to allow simultaneous solution of the approximately 45,000 nonlinear equations for the concentration of each of the glycan structures in each of the four compartments of the model.

Other parameters needed for the calculations, include compartment residence times, enzyme distributions between compartments, compartment volumes, total glycan concentration, and donor cosubstrate concentrations. These were estimated based on a variety of literature sources as detailed in our previous publications [19], [20]. It should be emphasized that these numbers are intended to be reasonable initial estimates subject to further refinement.

### Mapping glycan structure distributions to MALDI MS

Several steps are involved in generating the synthetic spectrum:

- The chemical formula of each model-predicted glycan structure is calculated after sample preparation. This step is necessary because after glycans are removed from their protein or lipid carrier; they are permethylated to improve the stability of the ions and reduce the variability of the mass spectrometer response factors of different glycans. This process replaces each OH group with an OCH_3_ group. The glycans also receive a sodium ion. These steps change the mass of the glycan necessitating this calculation.
- A table of isotope masses and abundances for each element is used to calculate the relative abundances and masses of the isotopic satellite peaks for each glycan. These follow a multinomial distribution.
- The model-predicted concentration of each glycan is multiplied by the relative abundances of each of its isotopic peaks and these are summed for all the glycans in the model.

The most significant isotope peaks for each glycan (those amounting to at least 10^−6^ of the total for the glycan) are calculated and stored in a database.

### Processing experimental mass spectra

The experimental MALDI mass spectra require processing before comparison with the synthetic mass spectra through baseline correction, mass calibration adjustment and peak integration.

The baseline correction method was adapted from Williams et al. (2005) [43]. The mass calibration was done by finding the linear mass adjustment that maximizes the sum of the experimental peaks interpolated to the theoretical masses of the model-predicted glycan peaks. An approximate area for each peak in the baseline-corrected and mass-calibrated spectrum was calculated as follows: First the nearest local maximum to the theoretical mass for each peak was determined to give a “peak height”. Then a “peak width” was determined for the 50 largest peaks by finding the point on either side of the maximum with an intensity of exp(-π/4) (or 45.6%) of the peak height. Note that multiplying this peak width by the peak height would give the exact area of a Guassian peak and also approximates the area of a skewed peak, such as a relatively narrow gamma distribution. The peak widths for the largest peaks so determined are then correlated as a linear function of peak molecular mass to accommodate the broadening of mass spectrometer peaks with increasing mass. The linear correlation of peak width vs. peak molecular mass is then used to calculate a peak width for every peak in the spectrum. The calculated peak width is multiplied by the peak height to estimate peak area. These peak areas are then normalized to add up to 100%. Examples of processed experimental spectra and calculated synthetic spectra are shown in Figure 3 and in Figure S1. The points on this plot are the area of each peak plotted against the mass at the peak maximum. Thus the curves on the plots are isotope envelopes.

**Figure 9:**
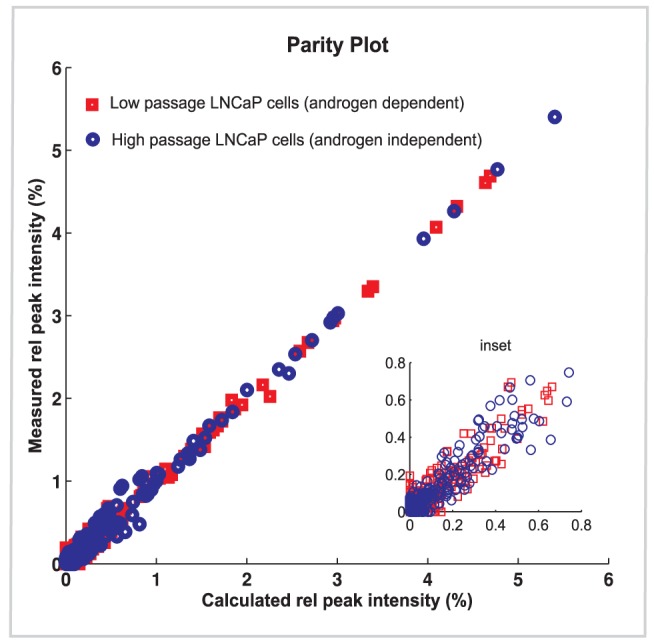
Parity plot. Shows fitting agreement between *projected* experimental mass spectra of glycans from high passage LNCaP cells (blue circle) and low passage LNCaP cells (red square) with synthetic mass spectra calculated from the model (RMS error 0.03). The comparison with *unprojected* experimental mass spectra is shown in Figure 2. Mass numbers from 1400 to 4000 were included.

After processing the experimental mass spectra still contain a significant number of minor peaks (actually isotopic satellite groups of peaks), which do not correspond to any glycans in the model. In most cases they do not correspond to any known N-glycans. Presumably these are artifacts of the sample processing, perhaps fragments produced in the mass spectrometer. In any event to avoid confounding of the model parameter adjustment step the preprocessed experimental spectra were further adjusted by projecting them onto only the masses contained in the model by means of a nonnegative linear regression method. This allows us to match the model parameters to only that part of the experimental mass spectrum explained by the model. However in visually comparing the model spectrum to the experimental spectrum, for example in Figures 2 and 3, the original unprojected experimental spectra have been used. Figure 9 shows the comparison of the model-generated mass spectra with the projected experimental spectra.

### Experimental data

Both experimental glycan structure measurements via MALDI TOF mass spectrometry and gene expression measurements using mRNA microarray are available at the Consortium of Functional Glycomics (CFG) website [22] and also in supplementary material Datasets S1, S2, and S3, which contains such results for a series of low, medium and high passage human prostate LNCaP cells. These cell types, with N-glycan mass spectra and the microarray data (MAEXP_291_040606) provided by Pi-Wan Cheng, represent a progression from androgen dependent cells to an androgen independent state. The human prostate cancer LNCaP C-33 and C-81 cell model used was established and characterized by [21]. C-33 cells include cells between passages 25 and 35, and C-81 cells include cells between passages 81 and 125.

### Gene expression data analysis

Three independent experimental mRNA data sets (MAEXP_291_040606- Dataset S3) for each of the high passage and low passage cell lines were used [22]. Expression levels were detected with the CFG Glycochip version 3, a custom designed gene chip that uses the Affymetrix technology and contains probe sets for over 1,000 human glycogenes. Each targeted mRNA transcript sequence was interrogated by 11 probe pairs of 25 base oligonucleotides each. Each probe pair consists of one perfect match (PM) - oligonucleotide complementary to a given portion of the targeted gene- and one mismatched (MM)-oligonucleotide identical in sequence to the PM probe, except for a single mismatched base-. The difference between the PM and MM probe signals among all probe pairs for a given gene was used to calculate the hybridization signal. This signal is a weighted average calculated for each probe set that represents the relative abundance of a transcript. In addition, a one-sided Wilcoxon Signed Rank Test is applied to this probe-pair intensity distribution to generate the p value. Thus, (p<0.04) is called present (P), p above 0.06 is called absent (A), and (0.04<p<0.06) is called marginal (M) [22].

## Supporting Information

Dataset S1 MALDI TOF glycan profiling for Low-passage human prostate LNCaP cells (LN-35).
(TXT)

Dataset S2 MALDI TOF glycan profiling for High passage Human prostate LNCaP cells (LN-130).
(TXT)

Dataset S3 Microarray screening of RNA samples from low, intermediate and high-passage human prostate LNCaP cells.
(XLS)

Figure S1 Annotated mass spectra for high and low passage LNCaP cells.
(PDF)

Figure S2 Parity plot assuming GnTV absent in the model.
(PDF)

Figure S3 Enzyme profile changes when GnTV is assumed absent for Low passage LNCaP cells.
(PDF)

Figure S4 Simplified representation of the sialylation mammalian pathway that indicates the synthesis of sugar nucleotide CMP-Neu5Ac (CMP-sialic acid).).
(PDF)

Table S1 Incorporation of gene expression into the model.
(PDF)

Table S2 Example of model reaction rules.
(PDF)

Table S3 LNCaP prostate cancer model kinetic network for enzyme processing of N-glycans. The kinetics of glycosylation was assumed to follow Michaelis-Menten as explained in Materials and Methods. The values of the kinetic parameters kf, Km, and Kmd for substrates and cosubstrates in each reaction are included.
(TXT)

Text S1 Details of model framework.
(PDF)

Text S2 Detecting incongruities in gene expression LNCaP cells data using the glycosylation model.
(PDF)

Text S3 CMP-Neu5Ac biosynthesis in high and low passage LNCaP cells.
(PDF)
