# Supplementary figures and images for: Integration of the Transcriptome and Glycome for Identification of Glycan Cell Signatures
Source: PLoS Comput Biol. 2013 Jan 10;9(1):e1002813. doi: 10.1371/journal.pcbi.1002813 (PMC3542073; doi:10.1371/journal.pcbi.1002813)

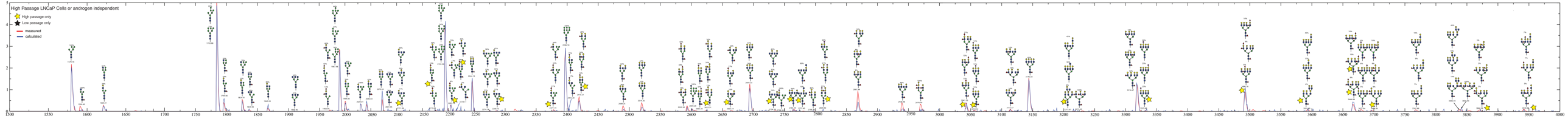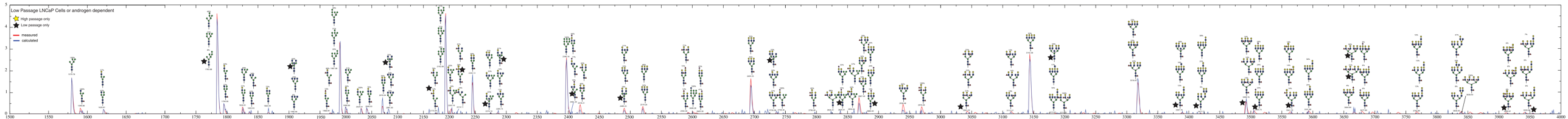

Supplement: Figure S1 — Annotated mass spectra for high and low passage LNCaP cells. (PDF) [file pcbi.1002813.s004.pdf]
