# Supplementary material for: Integration of the Transcriptome and Glycome for Identification of Glycan Cell Signatures
Source: PLoS Comput Biol. 2013 Jan 10;9(1):e1002813. doi: 10.1371/journal.pcbi.1002813 (PMC3542073; doi:10.1371/journal.pcbi.1002813)
Supplement: Figure S2 — Parity plot assuming GnTV absent in the model. (PDF) [file pcbi.1002813.s005.pdf]

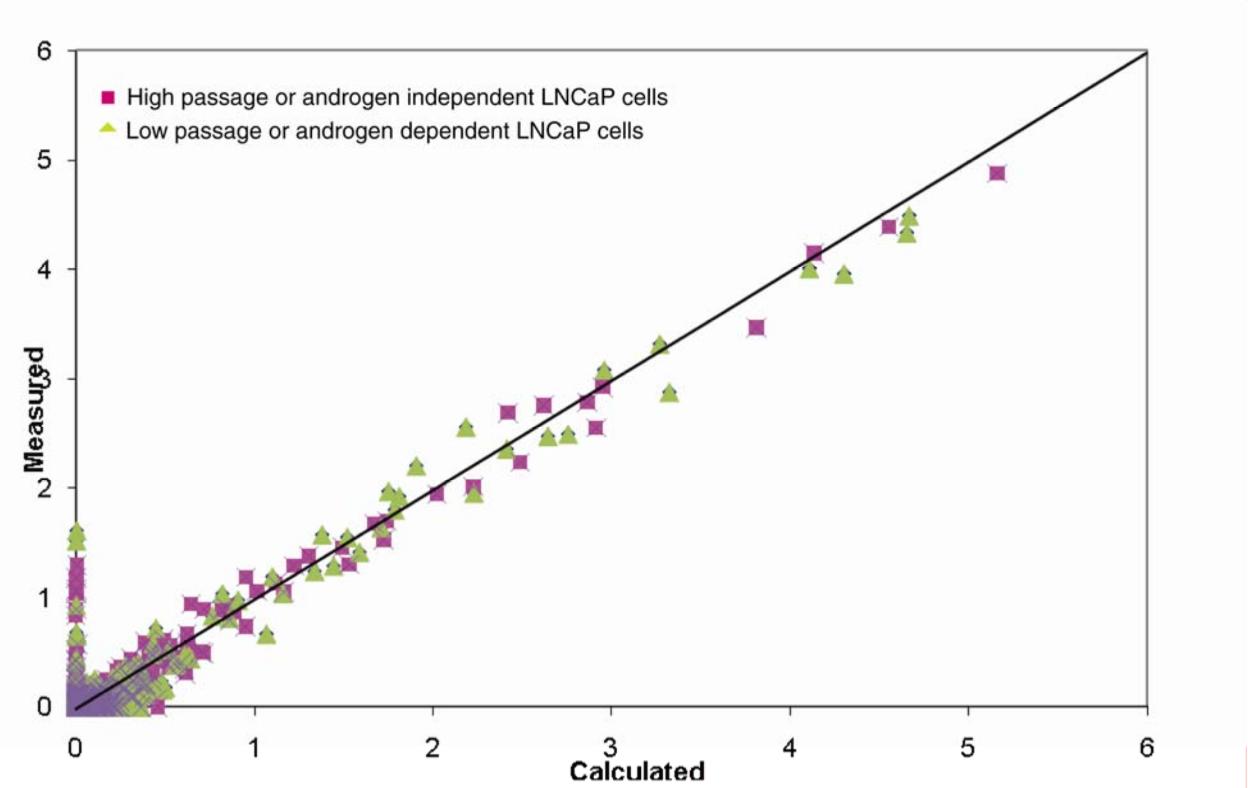

Figure S2: Parity plot assuming GnTV absent in the model. Comparison of measured mass spectra of glycans from LNCaP low passage prostate cancer human cells and LNCaP high passage prostate cancer human cells with synthetic mass spectra calculated from the model assuming GnTV absent. Mass numbers from 1400 to 4000 were included (RMS 0.08). The raw data files are available at the CFG web site and processed with software developed as described in Materials and Methods. (http://www.functionalglycomics.org/glycomics/publicdata/glycoprofiling.jsp).
