# Supplementary material for: Integration of the Transcriptome and Glycome for Identification of Glycan Cell Signatures
Source: PLoS Comput Biol. 2013 Jan 10;9(1):e1002813. doi: 10.1371/journal.pcbi.1002813 (PMC3542073; doi:10.1371/journal.pcbi.1002813)
Supplement: Figure S3 — Enzyme profile changes when GnTV is assumed absent for Low passage LNCaP cells. (PDF) [file pcbi.1002813.s006.pdf]

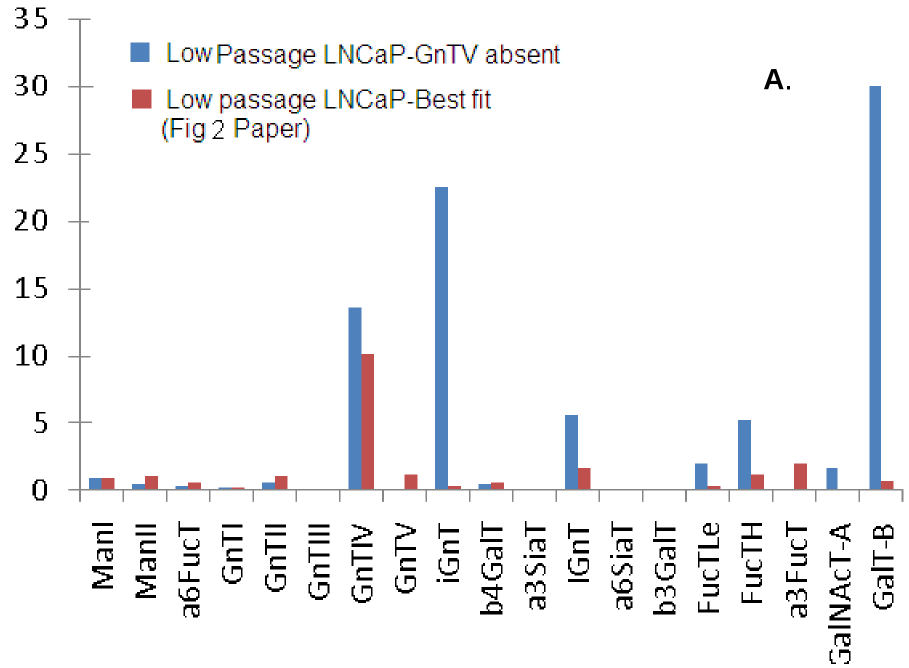

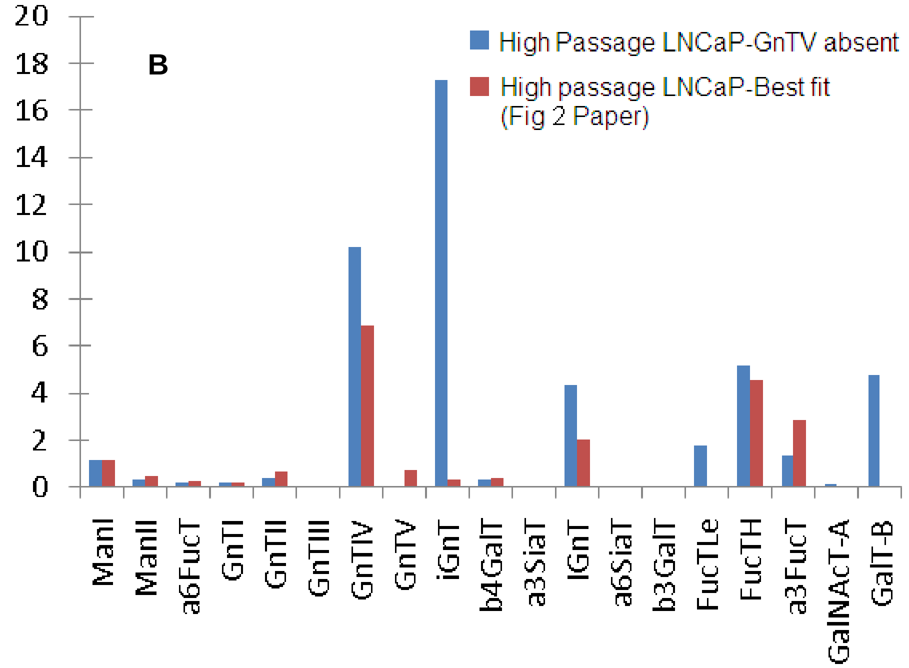

Figure S3 Enzyme profile changes when GnTV is assumed absent for Low passage LNCaP cells. Model predicted shifts in enzyme levels for panels A (low passage LNCaP cells) and B (high passage LNCaP cells). Comparison between the best fit case (Figure 2 in paper) with respect to the case assuming GnTV absent. The differences between both cases are expressed as enzymatic level shifts in % for all the enzymes in the model.
