# Supplementary material for: Integration of the Transcriptome and Glycome for Identification of Glycan Cell Signatures
Source: PLoS Comput Biol. 2013 Jan 10;9(1):e1002813. doi: 10.1371/journal.pcbi.1002813 (PMC3542073; doi:10.1371/journal.pcbi.1002813)
Supplement: Figure S4 — Simplified representation of the sialylation mammalian pathway that indicates the synthesis of sugar nucleotide CMP-Neu5Ac (CMP-sialic acid).). (PDF) [file pcbi.1002813.s007.pdf]

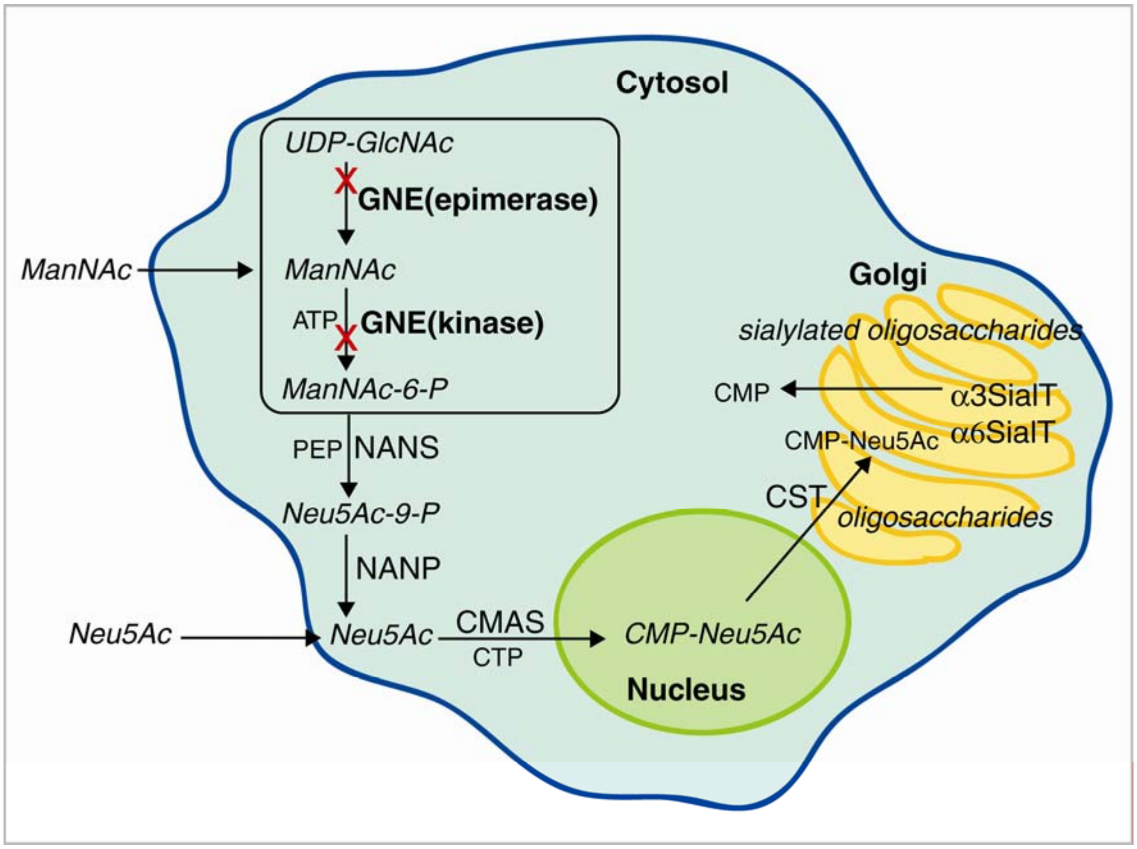

Figure S4 Simplified representation of the sialylation mammalian pathway that indicates the synthesis of sugar nucleotide CMP-Neu5Ac (CMP-sialic acid). The rectangle shows lack of expression of GNE in high and low passage LNCaP cell line from microarray data deposited at the Consortium of Functional Glycomics (http://www.functionalglycomics.org/glycomics/publicdata/glycoprofiling.jsp).
