# Supplementary material for: Integration of the Transcriptome and Glycome for Identification of Glycan Cell Signatures
Source: PLoS Comput Biol. 2013 Jan 10;9(1):e1002813. doi: 10.1371/journal.pcbi.1002813 (PMC3542073; doi:10.1371/journal.pcbi.1002813)
Supplement: Table S1 — Incorporation of gene expression into the model. (PDF) [file pcbi.1002813.s008.pdf]

Table S1. Model enzyme concentrations (μM) adjusted to match mass spectra of glycans from low and high passage human prostrate LNCaP cells while constraining enzyme concentration ratios to values derived from microarray expression levels.

Enzyme	Low passage LNCaP cells (μM)	High passage LNCaP cells (μM)	Model predicted ratio	Expression Ratio from microarray
ManI	3.41	4.28	1.25	
ManII	1.89	1.36	0.72	0.72
a6FucT	1.89	1.97	1.04	1.04
GnTI	0.86	1.05	1.22	1.22
GnTII	5.10	4.59	0.9	0.9
GnTIII	0.00	0.00		
GnTIV	0.64	0.49	0.76	0.76
GnTV	1.23	1.02	0.82	
iGnT	0.01	0.01	0.82	0.82
b4GalT	12.78	12.27	0.96	0.96
a3SiaT	0.0032	0.0027	0.81	0.81
IGnT	1.18	1.00	0.85	0.85
a6SiaT	0.00	0.11		
b3GalT	0.00	0.00		
FucTLe	0.00	0.00	1.5	1.5
FucTH	0.019	0.032	1.72	1.72
a3FucT	0.032	0.034	1.07	1.07
GalNAcT-A	0.00	0.00		
GalT-B	0.00	0.00		
