# Supplementary material for: Integration of the Transcriptome and Glycome for Identification of Glycan Cell Signatures
Source: PLoS Comput Biol. 2013 Jan 10;9(1):e1002813. doi: 10.1371/journal.pcbi.1002813 (PMC3542073; doi:10.1371/journal.pcbi.1002813)

Table S2:Example of model reaction rules

Enzyme	Substrate	Product	Constrain
GNTV	(GNb2Ma3	(GNb2(GNb4)Ma3	~Gnbis

GNb2Ma3(GNb2Ma6)Mb4GNb4GN;Asn →

GNb2(GNb4)Ma3(GNb2Ma6)Mb4GNb4GN;Asn

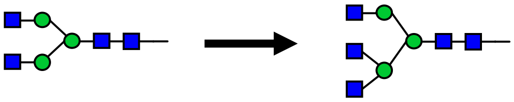

GNb2Ma3(Ma3(Ma6)Ma6)Mb4GNb4(Fa6)GN;Asn →

GNb2(GNb4)Ma3(Ma3(Ma6)Ma6)Mb4GNb4(Fa6)GN;Asn

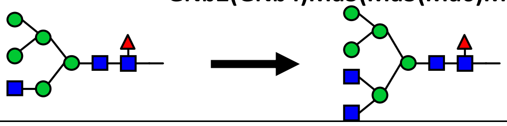

Supplement: Table S2 — Example of model reaction rules. (PDF) [file pcbi.1002813.s009.pdf]
