# Supplementary material for: Integration of the Transcriptome and Glycome for Identification of Glycan Cell Signatures
Source: PLoS Comput Biol. 2013 Jan 10;9(1):e1002813. doi: 10.1371/journal.pcbi.1002813 (PMC3542073; doi:10.1371/journal.pcbi.1002813)
Supplement: Text S1 — Details of model framework. (PDF) [file pcbi.1002813.s011.pdf]

Details of model framework

The current framework to represent glycan structures was explained in Materials and Methods. Enzyme specificities are denoted using a string substitution methodology. The basic idea is to express the function of an enzyme by a pair of character strings. The first, or substrate substring (Table 2-column 3), is the formula representation of the substructure of a glycan that the enzyme can act upon. The second, or product substring (table 2-column 4), is the formula representation of the substructure that the substrate substructure is transformed into by the enzyme action. Additional constraints on the substrate (Table 2-column 5), that are required for the enzyme action are expressed as a set of logical tests for the presence or absence of other substrings in the complete substrate formula. For example this rule results in many reactions; in Table S2 we show an example for two of these reactions.

The software developed in the project takes Tables 2, 4 and 5 as input, along with a starting structure, and generates all possible structures and reactions for the selected list of enzymes. While the prototype model generated 10, 000 glycans (Table S3), the extended model generates hundreds of thousands of structures with millions of reactions. Indeed, without special termination rules, the number of possible structures is infinite. However the model size is kept manageable by setting a maximum molecular weight for the glycan products. In addition a method has been developed to estimate the abundance of the glycans during the network generation phase so that structures of negligible abundance can be dropped. These methods result in reasonably sized models, with 10,000 – 25,000 structures.
