# Supplementary material for: Integration of the Transcriptome and Glycome for Identification of Glycan Cell Signatures
Source: PLoS Comput Biol. 2013 Jan 10;9(1):e1002813. doi: 10.1371/journal.pcbi.1002813 (PMC3542073; doi:10.1371/journal.pcbi.1002813)
Supplement: Text S2 — Detecting incongruities in gene expression LNCaP cells data using the glycosylation model. (PDF) [file pcbi.1002813.s012.pdf]

Detecting incongruities in gene expression LNCaP cells data using the glycosylation model

Although the microarray experiments analyzed here provide high-quality data, artifacts and limitations should be addressed. In this section we will extend the discussion in the paper on how the model was used to detect incongruities in the gene expression data. In particular we will exemplify by focusing in one main incongruity found using the glycosylation model: the existence of an inactive gene probe MGAT5 for GnTV in the CFG glycochip version 3. Other incongruities which are not discussed here were also identified by the model such as absence of the ManI probe in the glycochip V3 of CFG. In the microarray, the genes for GnTV on Glycochip version 3 from CFG used in the current studies are MGAT5 and MGAT5B. The expression of MGAT5 for both cell types and MGAT5B for the high passage cells are interpreted as absent according to the microarray data. The expression level of MGAT5B is indicated as present but only marginally so for the low passage cells. Interestingly, processing of MALDI TOF mass spectra data by the glycosylation model indicates the presence GnTV (MAGAT5) which diverges from the absence of their signals in the gene expression data. By assuming into the model, the corresponding gene expression outputs for GnTV (MAGAT5).

The model allowed evaluating the impact of removing GnTV. This was assessed by evaluating how the model fitting to the experimental data is affected after removing GnTV. A measure of model agreement with experimental data can be indicated by calculating the Root-mean-square (RMS). RMS errors show how well the glycosylation model predicts the mass spectra data. The lower the RMS error the better the model fitting to the experimental data. For example, if the RMS value is large, the difference between the experimental data and the model is large. However if the RMS is small the model predicts well the experimental mass spectra data.

Effect of GnTV absence in high and low passage LNCaP cells

GnTV enzyme level was assumed absent in the glycosylation model to agree with the marginal levels of GnTV in the expression data. The model predictions show that this scenario results in a non optimal model solution. Indication of that is given by the increase in RMS from 0.05 -corresponding to the best fit Figure 2 in paper- to RMS of 0.08 -corresponding to Figure S2. Here we observe an increased dispersion of the data, pointing to a non optimal solution. In Figures S3 we show how absence of GnTV has affected the level of the other enzymes present in the model as compared to the best fit case corresponding to Figure 2 in the paper.
