# Supplementary material for: Integration of the Transcriptome and Glycome for Identification of Glycan Cell Signatures
Source: PLoS Comput Biol. 2013 Jan 10;9(1):e1002813. doi: 10.1371/journal.pcbi.1002813 (PMC3542073; doi:10.1371/journal.pcbi.1002813)
Supplement: Text S3 — CMP-Neu5Ac biosynthesis in high and low passage LNCaP cells. (PDF) [file pcbi.1002813.s013.pdf]

CMP-Neu5Ac biosynthesis in high and low passage LNCaP cells

The sialylation of N-glycans requires the transfer of the sialic acid (Neu5Ac) group from the nucleotide sugar CMP-Neu5Ac (CMP-sialic acid) onto the oligosaccharide acceptor ending in a Galactose (Gal) residue through α2,3- or α2,6-linkage. Thus successful sialylation of N-glycans requires not only the activity of a sialyltransferase but also the presence of a CMP-Neu5Ac nucleotide sugar. The CMP-Neu5Ac is generated through the biosynthetic pathway shown in (Figure S4). Critical steps of CMP-Neu5Ac biosynthesis are the two sequential reactions catalyzed by the bifunctional UDP-N-acetylglucosamine-2-epimerase/N-acetylmannosamine kinase (GNE) enzyme in the cytosol (indicated by rectangle in Figure S4). These reactions are the initial epimerization of UDP-N-acetylglucosamine (UDP-GlcNAc) to N-acetylmannosamine (ManNAc), and the phosphorylation to N-acetylmannosamine-6-phosphate (ManNAc-6-P). Further enzymatic reactions convert the ManNAc-6-P into Neu5Ac followed by the addition of CTP in the nucleus to generate CMP-NeuAc. The genes associated with sialic acid biosynthesis are also available on the glycochip version 3 chip. Interestingly, the transcript levels for GNE in the microarray for the high and low passage LNCaP are interpreted as absent for the microarray. In Figure S4, **t**he reactions associated to GNE for high and low passage LNCaP cells are indicated within the square shape. The fact that the microarray data shows some critical enzymes in the pathway for sialic acid precursor processing as absent, may have influence in post processing sialylation in the Golgi. So even if the sialyl transferases enzymes show as present in the microarray data, the sialic acid precursor synthesis is affected and the model outputs based on structural data seem to reflect that.

**Abbreviations**	
NANP	NeuAc 9-*phosphate* phosphatase
CMAS	CMP-NeuAc synthetase
CST	cytidine monophosphate-sialic acid transporter
ST	Sialyl Transferase
ATP	Adenosine triphosphate
PEP	Phosphoenolpyruvate
CMP	Cytosine Monophosphate
CTP	Cytidine 5’-triphosphate
